# Genomics dataset of unidentified disclosed isolates

**DOI:** 10.1016/j.dib.2016.06.010

**Published:** 2016-06-15

**Authors:** Bhagwan N. Rekadwad

**Affiliations:** School of Life Sciences, Swami Ramanand Teerth Marathwada University, Nanded, Maharashtra 431606, India

**Keywords:** BioLABs, Blunt ends, Genomics, NEB cutter, Restriction digestion, Short DNA sequences, Sticky ends

## Abstract

Analysis of DNA sequences is necessary for higher hierarchical classification of the organisms. It gives clues about the characteristics of organisms and their taxonomic position. This dataset is chosen to find complexities in the unidentified DNA in the disclosed patents. A total of 17 unidentified DNA sequences were thoroughly analyzed. The quick response codes were generated. AT/GC content of the DNA sequences analysis was carried out. The QR is helpful for quick identification of isolates. AT/GC content is helpful for studying their stability at different temperatures. Additionally, a dataset on cleavage code and enzyme code studied under the restriction digestion study, which helpful for performing studies using short DNA sequences was reported. The dataset disclosed here is the new revelatory data for exploration of unique DNA sequences for evaluation, identification, comparison and analysis.

**Specifications** **Table**

**Table:** 

Subject area	*Life Sciences*
More specific subject area	*Microbiology, Genomics, Bioinformatics, Bacterial Systematics*
Type of data	*Table, Figures*
How data was acquired	*Through NCBI BioSample database*
Data format	*Raw and Analyzed*
Experimental factors	*Dataset obtained through bioinformatics tool*
Experimental features	*Only disclosed genome sequences were used*
Data source location	School of Life Sciences, S. R. T. M. University, Nanded, India
Data accessibility	Data available within article and via the *NCBI repository*http://www.ncbi.nlm.nih.gov/nuccore.

**Value of the data**

• Data provides information of the AT and GC percentage of unidentified isolates.
• This data would be valuable for qualitative and quantitative analysis newly isolated and unidentified strains.
• This data provides exact position of restriction sites to create blunt and sticky ends and gives an idea about cleavage affected by methylation.

## 1 Data

This paper contains data on data for QR codes, GC percentage and DNA sequence analysis of 17 unidentified strains. Genome sequences of unidentified bacterial strains which were disclosed from the patents US 6596510 and WO 9906567 were retrieved in FASTA format via NCBI nuccore database. These downloaded sequences were used to create quick response (QR) codes and digitized using ENDMEMO GC calculating and GC plotting tool. The AT and GC percentage, number of cleavage code (blunt end, 5′ and 3′ sticky ends) and number of enzyme code (cleavage affected methylation) were determined using BioLabs NEB cutter tool (NEW ENGLAND BioLabs. Inc. https://www.neb.com/).

## 2 Experimental design, materials and methods

**Fig. 1:**
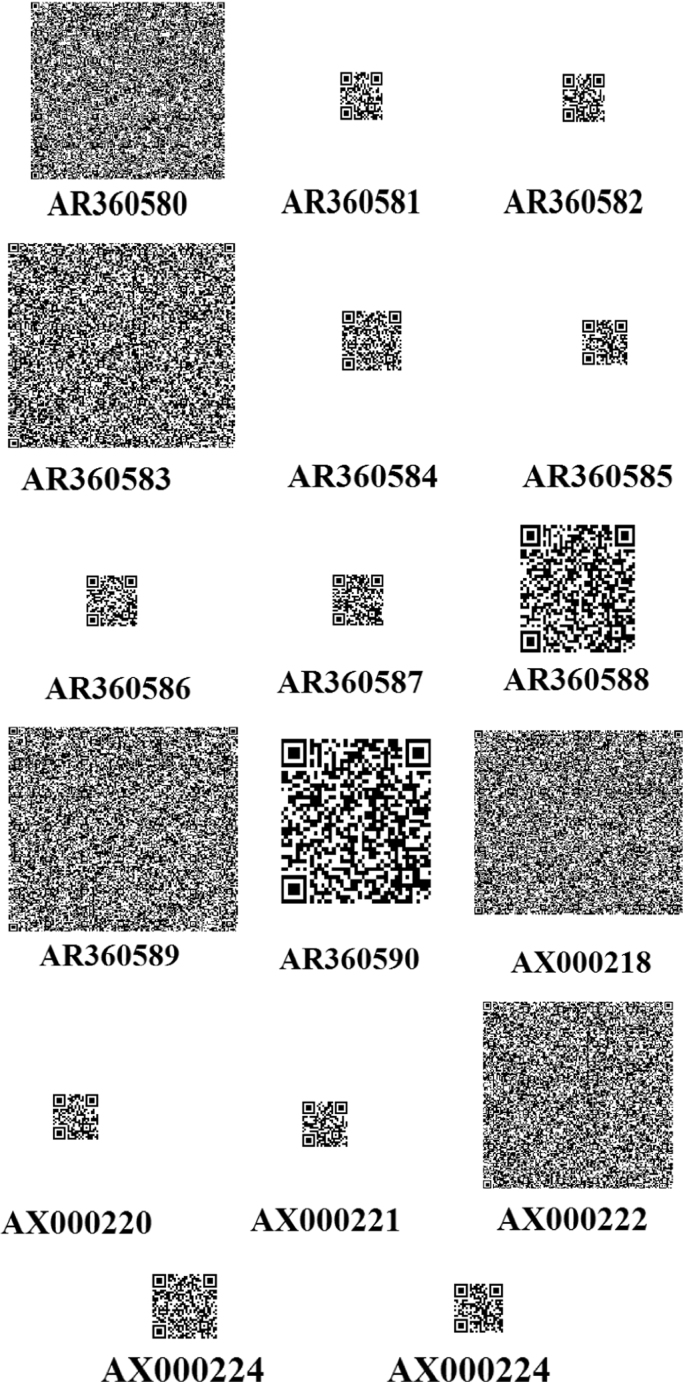
QR codes of unidentified sequences (AR360580-AR360590, AX000218, AX000220, AX000221, AX000222, AX000224 and AX000225).

**Fig. 2:**
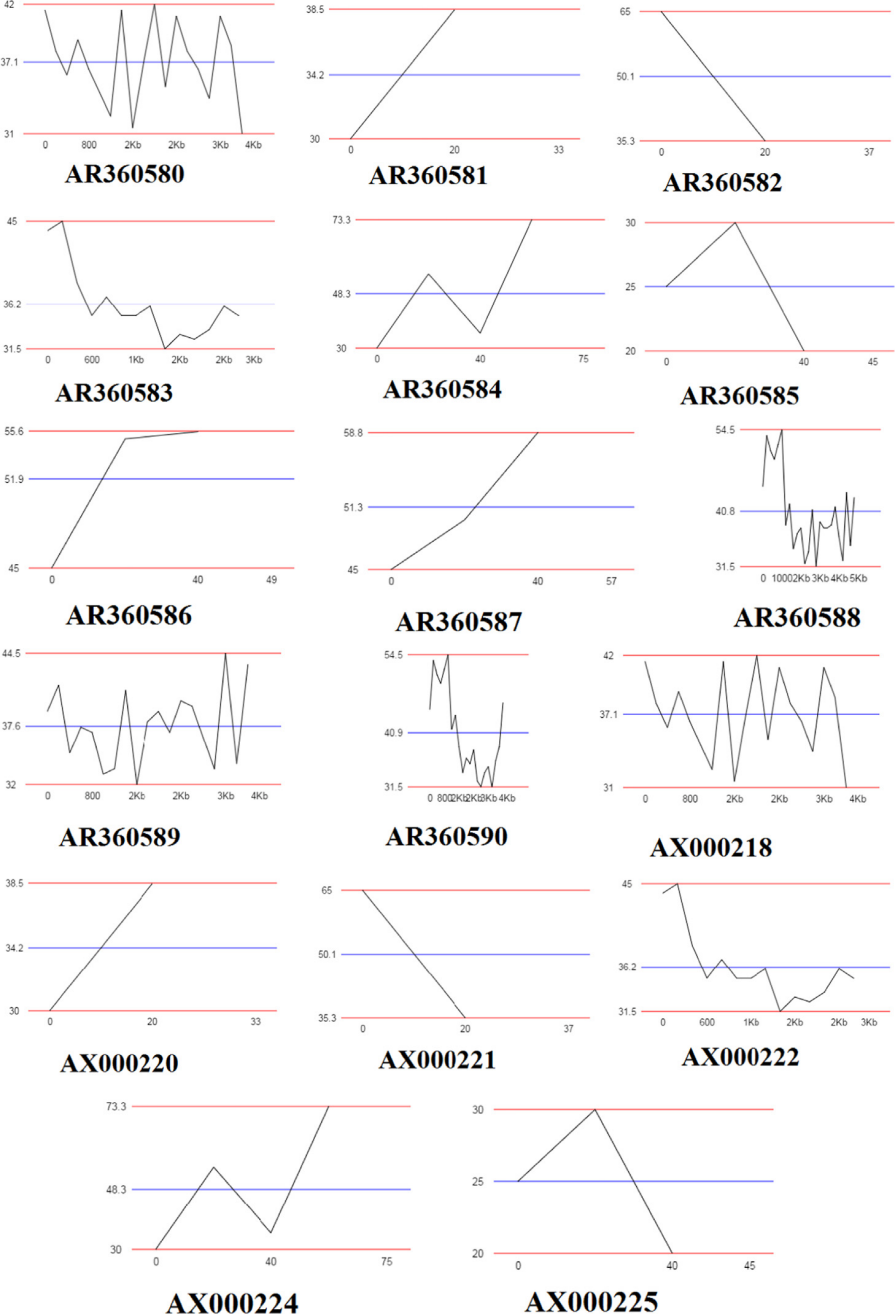
GC plot of unidentified sequences (AR360580-AR360590, AX000218, AX000220, AX000221, AX000222, AX000224 and AX000225).

**Fig. 3:**
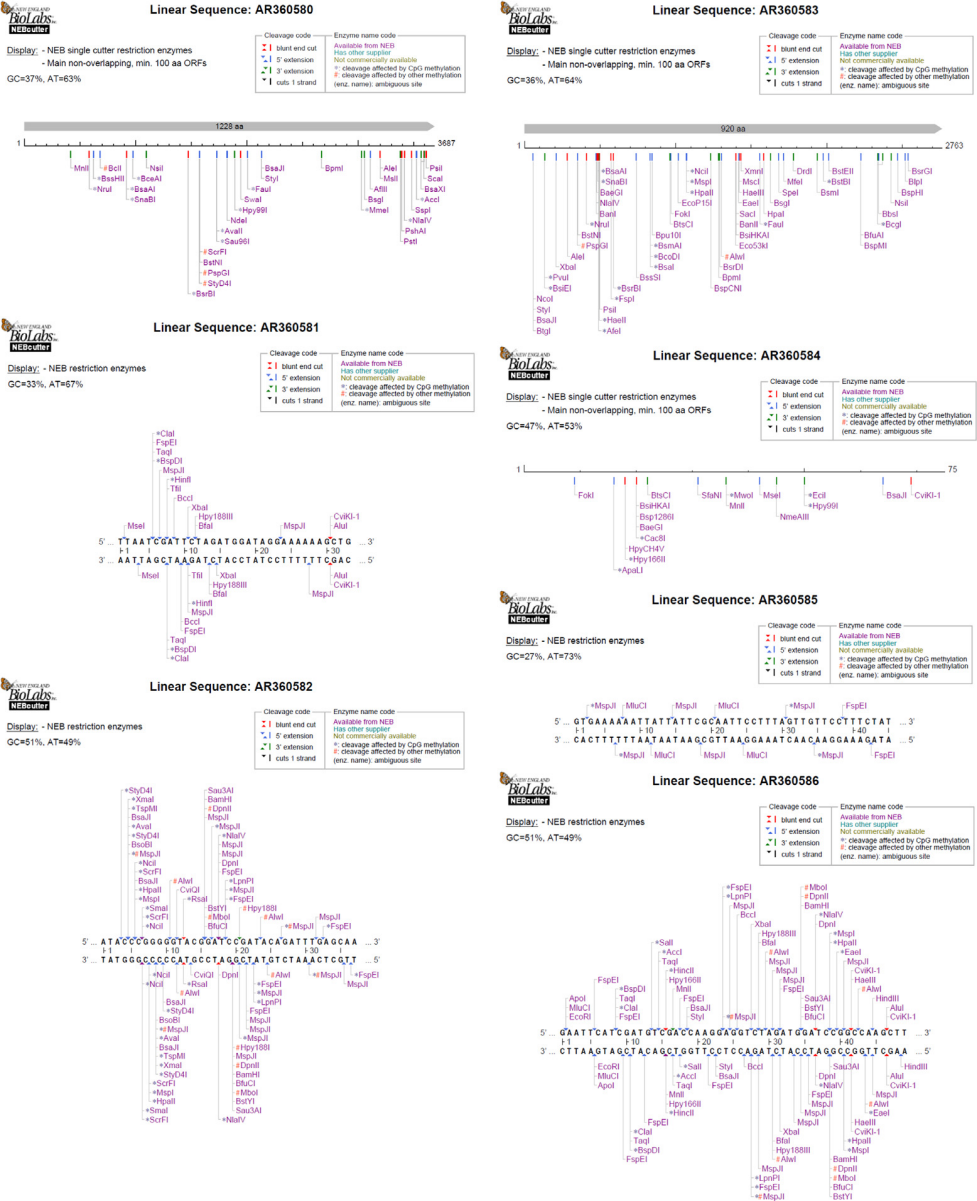

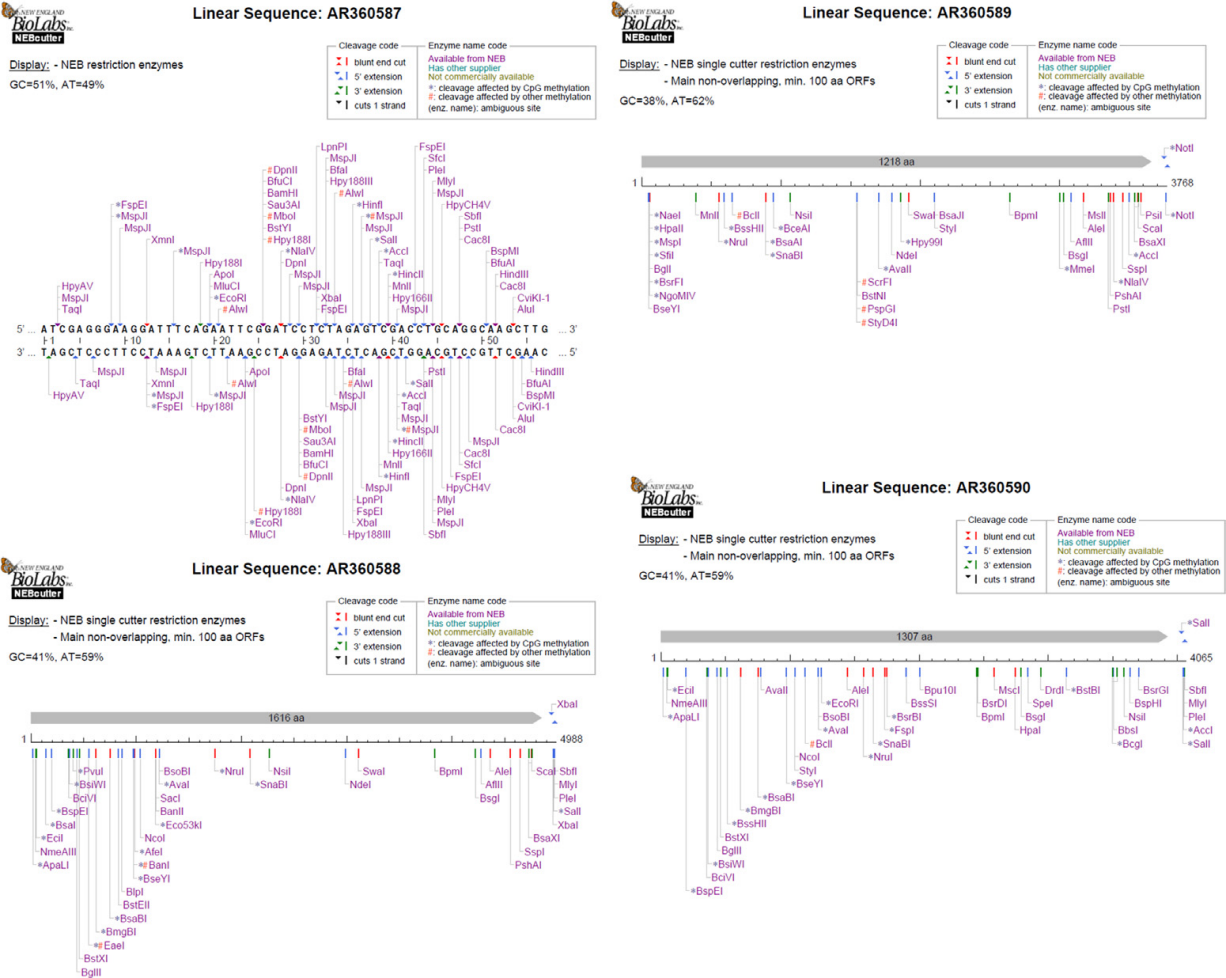

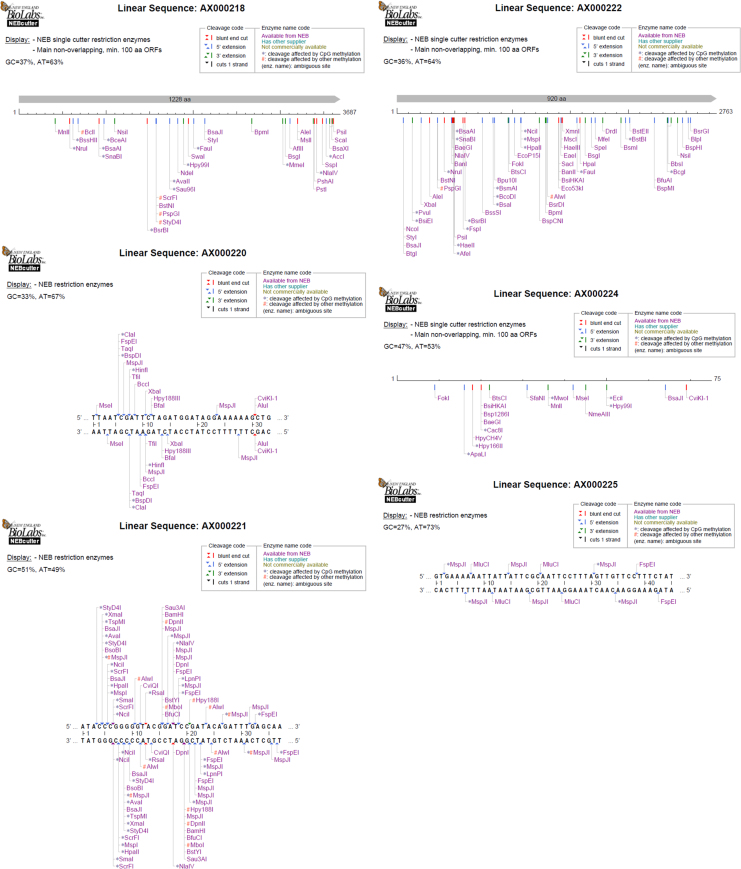
NEB restriction enzyme digestion of unidentified sequences of patents (Accession No.: AR360580-AR360590, AX000218, AX000220, AX000221, AX000222, AX000224 and AX000225).

A total of 17 genome sequences of disclosed unidentified bacteria (*AR360580, AR360581, AR360582, AR360583, AR360584, AR360585, AR360586, AR360587, AR360588, AR360589, AR360590, AX000218, AX000220, AX000221, AX000222, AX000224 and AX000225*) were saved in FASTA format via NCBI BioSample DNA database. DNABarID tool was used for creation of QR codes (Fig. 1). ENDMEMO GC calculating and GC plotting tool was used to determine percentage of nucleotides in the genome. Pattern of GC distribution in complete DNA sequence showed through graphical representations in Fig. 2. Upper and lower red line indicate maximum and minimum percentage of GC content distribution in complete DNA sequence, while middle blue line indicates average GC percentage [1], [2], [3], [4], [5], [6]. NEB cutter tool was used analysis of DNA sequence of unidentified isolates. The number of cleavage to possible in the form of blunt end, 5′ and 3′ sticky ends was determined. The number of enzyme codes was determined. It gives exact information about cleavage affected CpG methylation and other types of methylation possible caused by biomolcules. Additionally, BioLabs database determined the AT and GC percentage in the genome [7], [8] (Fig. 3; Table 1).

## Figures and Tables

**Table 1:** Unidentified sequences: Genomic analysis and restriction digestion using NEB single cutter restriction enzymes.

S. N.	Accession number	Name of sequence	Maximum GC%	Average GC%	Average AT%	Number of cleavage code			Number of enzyme code	
						Blunt end cut	5′ extension	3′ extension	*: cleavage affected by CpG methylation	#: cleavage affected by other methylation
1	AR360580	Sequence 1 from patent US 6596510	42	37	63	10	10	9	13	4
2	AR360581	Sequence 3 from patent US 6596510	38.5	22	67	1	7	–	3	–
3	AR360582	Sequence 4 from patent US 6596510	65	51	49	2	12	2	20	7
4	AR360583	Sequence 5 from patent US 6596510	45	36	64	11	11	14	17	2
5	AR360584	Sequence 7 from patent US 6596510	73.37	47	53	3	5	4	6	–
6	AR360585	Sequence 8 from patent US 6596510	30	27	73	–	6	–	2	–
7	AR360586	Sequence 9 from patent US 6596510	55.6	51	49	4	20	1	12	5
8	AR360587	Sequence 10 from patent US 6596510	58.8	51	49	5	18	4	10	6
9	AR360588	Sequence 11 from patent US 6596510	54.5	41	59	11	15	10	17	2
10	AR360589	Sequence 12 from patent US 6596510	44.5	38	62	9	12	10	17	4
11	AR360590	Sequence 13 from patent US 6596510	54.5	41	59	9	19	11	18	1
12	AX000218	Sequence 1 from Patent WO 9906567	42	37	63	10	11	9	13	4
13	AX000220	Sequence 3 from patent WO 9906567	38.5	33	67	1	8	–	3	7
14	AX000221	Sequence 4 from patent WO 9906567	65	51	49	2	12	1	21	7
15	AX000222	Sequence 5 from patent WO 9906567	45	36	64	11	23	12	18	2
16	AX000224	Sequence 7 from patent WO 9906567	73.3	47	53	3	5	4	6	–
17	AX000225	Sequence 8 from patent WO 9906567	30	27	73	–	6	–	2	–
