# Supplementary figures and images for: Genomics dataset of unidentified disclosed isolates
Source: Data Brief. 2016 Jun 15;8:579–87. doi: 10.1016/j.dib.2016.06.010 (PMC4930343; doi:10.1016/j.dib.2016.06.010)

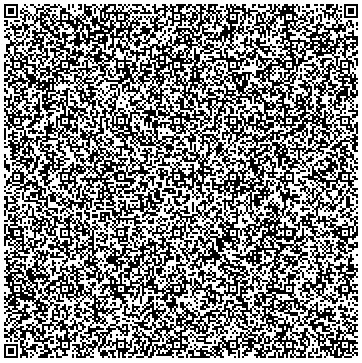

Supplement: Supplementary file 1 — Supplementary material [file mmc1.zip › QR Codes Unidentified sequences/AR360580.jpg]

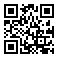

Supplement: Supplementary file 1 — Supplementary material [file mmc1.zip › QR Codes Unidentified sequences/AR360581.jpg]

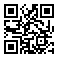

Supplement: Supplementary file 1 — Supplementary material [file mmc1.zip › QR Codes Unidentified sequences/AR360582.jpg]

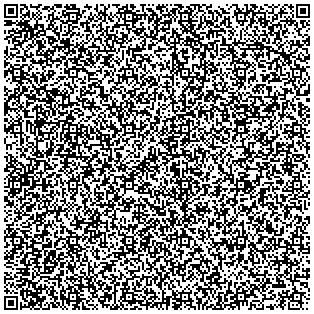

Supplement: Supplementary file 1 — Supplementary material [file mmc1.zip › QR Codes Unidentified sequences/AR360583.jpg]

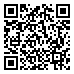

Supplement: Supplementary file 1 — Supplementary material [file mmc1.zip › QR Codes Unidentified sequences/AR360584.jpg]

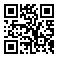

Supplement: Supplementary file 1 — Supplementary material [file mmc1.zip › QR Codes Unidentified sequences/AR360585.jpg]

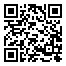

Supplement: Supplementary file 1 — Supplementary material [file mmc1.zip › QR Codes Unidentified sequences/AR360586.jpg]

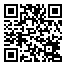

Supplement: Supplementary file 1 — Supplementary material [file mmc1.zip › QR Codes Unidentified sequences/AR360587.jpg]

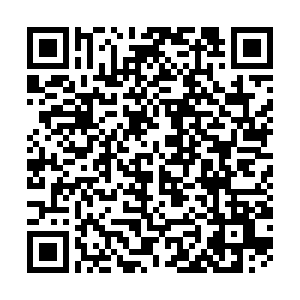

Supplement: Supplementary file 1 — Supplementary material [file mmc1.zip › QR Codes Unidentified sequences/AR360588.png]

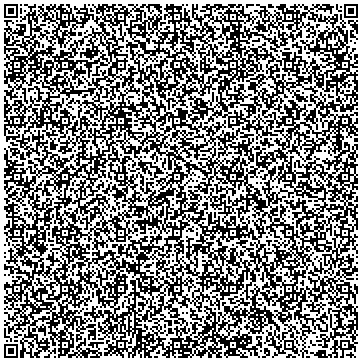

Supplement: Supplementary file 1 — Supplementary material [file mmc1.zip › QR Codes Unidentified sequences/AR360589.jpg]

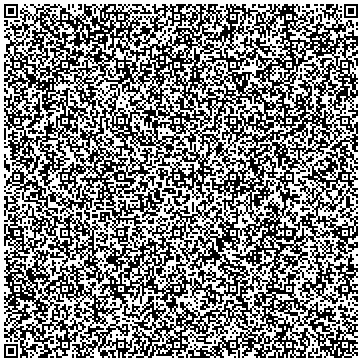

Supplement: Supplementary file 1 — Supplementary material [file mmc1.zip › QR Codes Unidentified sequences/AX000218.jpg]

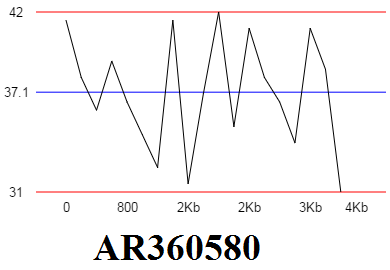

Supplement: Supplementary file 2 — Supplementary material [file mmc2.zip › GC plot of unidentified bacteria/AR360580.png]

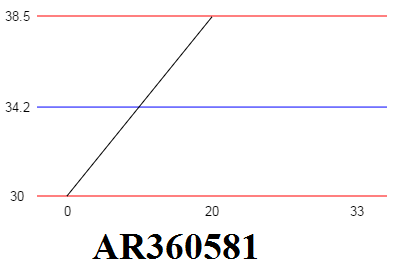

Supplement: Supplementary file 2 — Supplementary material [file mmc2.zip › GC plot of unidentified bacteria/AR360581.png]

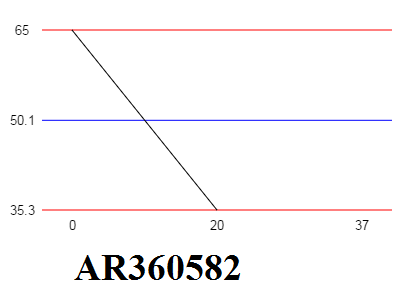

Supplement: Supplementary file 2 — Supplementary material [file mmc2.zip › GC plot of unidentified bacteria/AR360582.png]

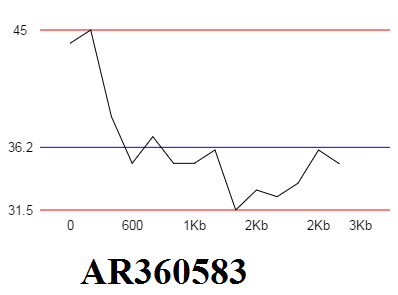

Supplement: Supplementary file 2 — Supplementary material [file mmc2.zip › GC plot of unidentified bacteria/AR360583.png]

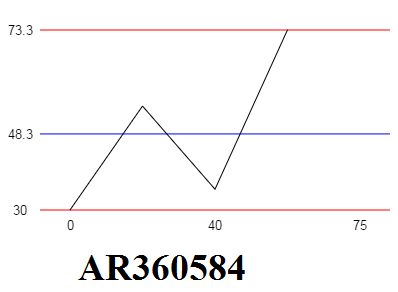

Supplement: Supplementary file 2 — Supplementary material [file mmc2.zip › GC plot of unidentified bacteria/AR360584.png]

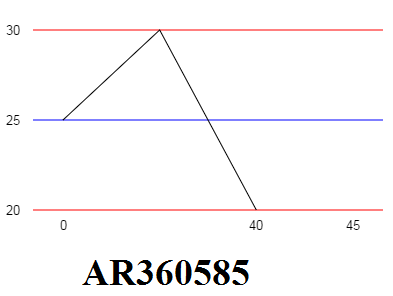

Supplement: Supplementary file 2 — Supplementary material [file mmc2.zip › GC plot of unidentified bacteria/AR360585.png]

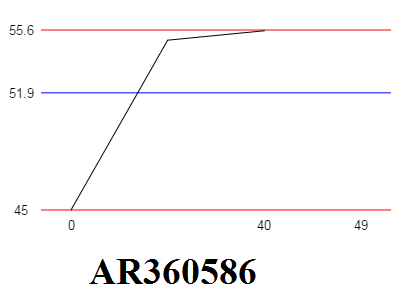

Supplement: Supplementary file 2 — Supplementary material [file mmc2.zip › GC plot of unidentified bacteria/AR360586.png]

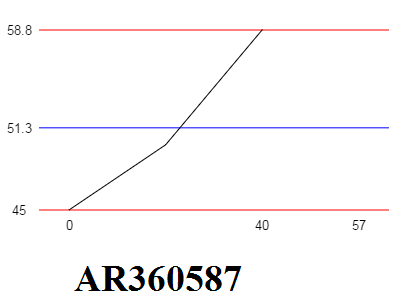

Supplement: Supplementary file 2 — Supplementary material [file mmc2.zip › GC plot of unidentified bacteria/AR360587.png]

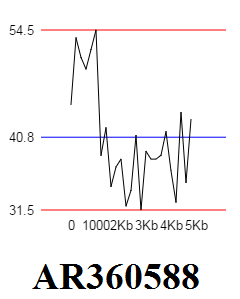

Supplement: Supplementary file 2 — Supplementary material [file mmc2.zip › GC plot of unidentified bacteria/AR360588.png]

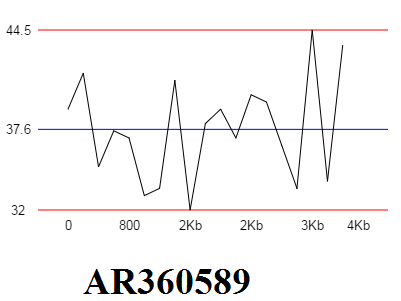

Supplement: Supplementary file 2 — Supplementary material [file mmc2.zip › GC plot of unidentified bacteria/AR360589.png]

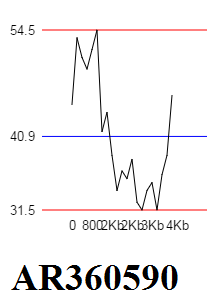

Supplement: Supplementary file 2 — Supplementary material [file mmc2.zip › GC plot of unidentified bacteria/AR360590.png]

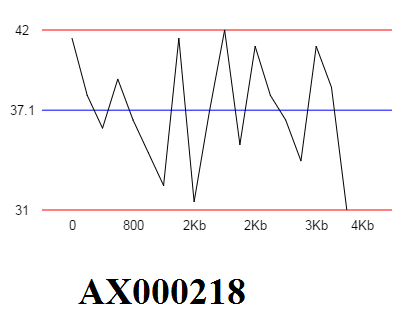

Supplement: Supplementary file 2 — Supplementary material [file mmc2.zip › GC plot of unidentified bacteria/AX000218.png]

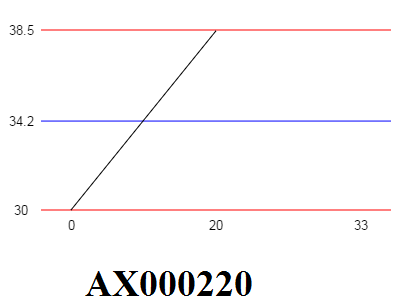

Supplement: Supplementary file 2 — Supplementary material [file mmc2.zip › GC plot of unidentified bacteria/AX000220.png]

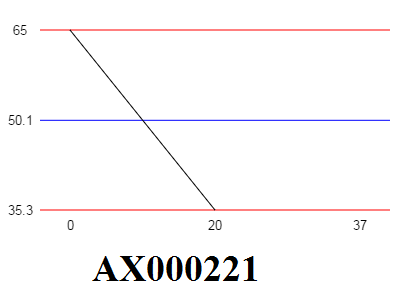

Supplement: Supplementary file 2 — Supplementary material [file mmc2.zip › GC plot of unidentified bacteria/AX000221.png]

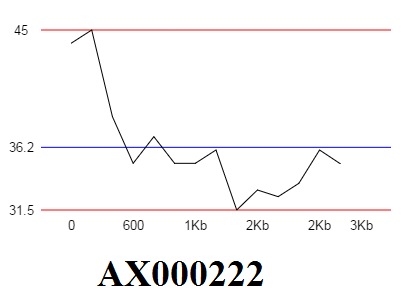

Supplement: Supplementary file 2 — Supplementary material [file mmc2.zip › GC plot of unidentified bacteria/AX000222.png]

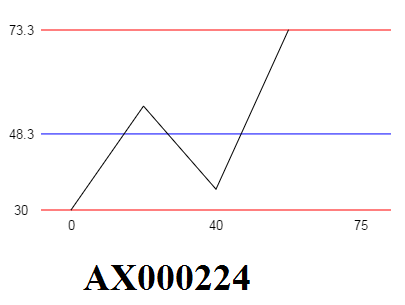

Supplement: Supplementary file 2 — Supplementary material [file mmc2.zip › GC plot of unidentified bacteria/AX000224.png]

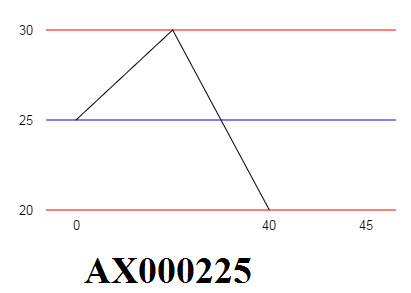

Supplement: Supplementary file 2 — Supplementary material [file mmc2.zip › GC plot of unidentified bacteria/AX000225.png]
