# Supplementary material for: Genomics dataset of unidentified disclosed isolates
Source: Data Brief. 2016 Jun 15;8:579–87. doi: 10.1016/j.dib.2016.06.010 (PMC4930343; doi:10.1016/j.dib.2016.06.010)

Linear Sequence: AR360580

Display: - NEB single cutter restriction enzymes
- Main non-overlapping, min. 100 aa ORFs

GC=37%, AT=63%

Cleavage code: blunt end cut; 5' extension; 3' extension; cuts 1 strand

Enzyme name code: Available from NEB; Has other supplier; Not commercially available; *: cleavage affected by CpG methylation; #: cleavage affected by other methylation; (enz. name): ambiguous site

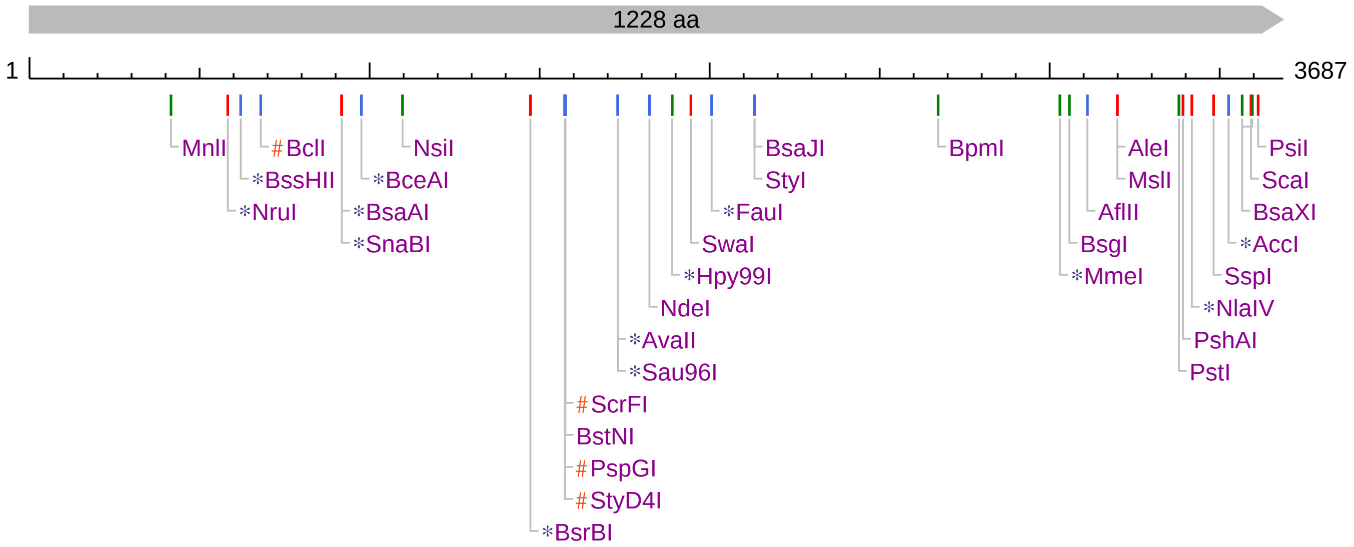

Supplement: Supplementary file 3 — Supplementary material [file mmc3.zip › AR360580 BioLab NEBcutter result.pdf]
