# Supplementary material for: Genomics dataset of unidentified disclosed isolates
Source: Data Brief. 2016 Jun 15;8:579–87. doi: 10.1016/j.dib.2016.06.010 (PMC4930343; doi:10.1016/j.dib.2016.06.010)

Linear Sequence: AR360582

NEW ENGLAND BioLabs Inc.
NEBcutter

Display: - NEB restriction enzymes

GC=51%, AT=49%

Cleavage code
- blunt end cut
- 5' extension
- 3' extension
- cuts 1 strand

Enzyme name code
- Available from NEB
- Has other supplier
- Not commercially available
- *: cleavage affected by CpG methylation
- #: cleavage affected by other methylation
- (enz. name): ambiguous site

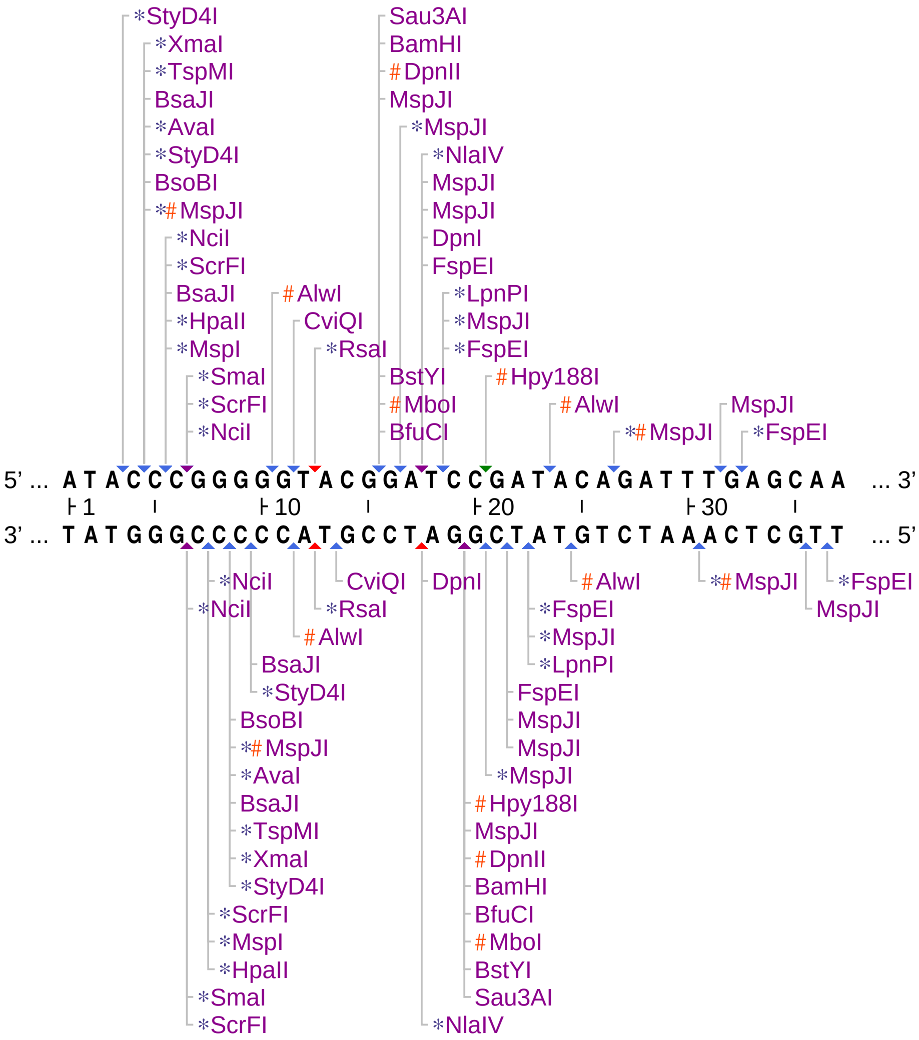

Supplement: Supplementary file 3 — Supplementary material [file mmc3.zip › AR360582 BioLab NEBcutter result.pdf]
