# Supplementary material for: Genomics dataset of unidentified disclosed isolates
Source: Data Brief. 2016 Jun 15;8:579–87. doi: 10.1016/j.dib.2016.06.010 (PMC4930343; doi:10.1016/j.dib.2016.06.010)

Linear Sequence: AR360583

Display: - NEB single cutter restriction enzymes
- Main non-overlapping, min. 100 aa ORFs

GC=36%, AT=64%

Cleavage code
- blunt end cut
- 5' extension
- 3' extension
- cuts 1 strand

Enzyme name code
Available from NEB
Has other supplier
Not commercially available
*: cleavage affected by CpG methylation
#: cleavage affected by other methylation
(enz. name): ambiguous site

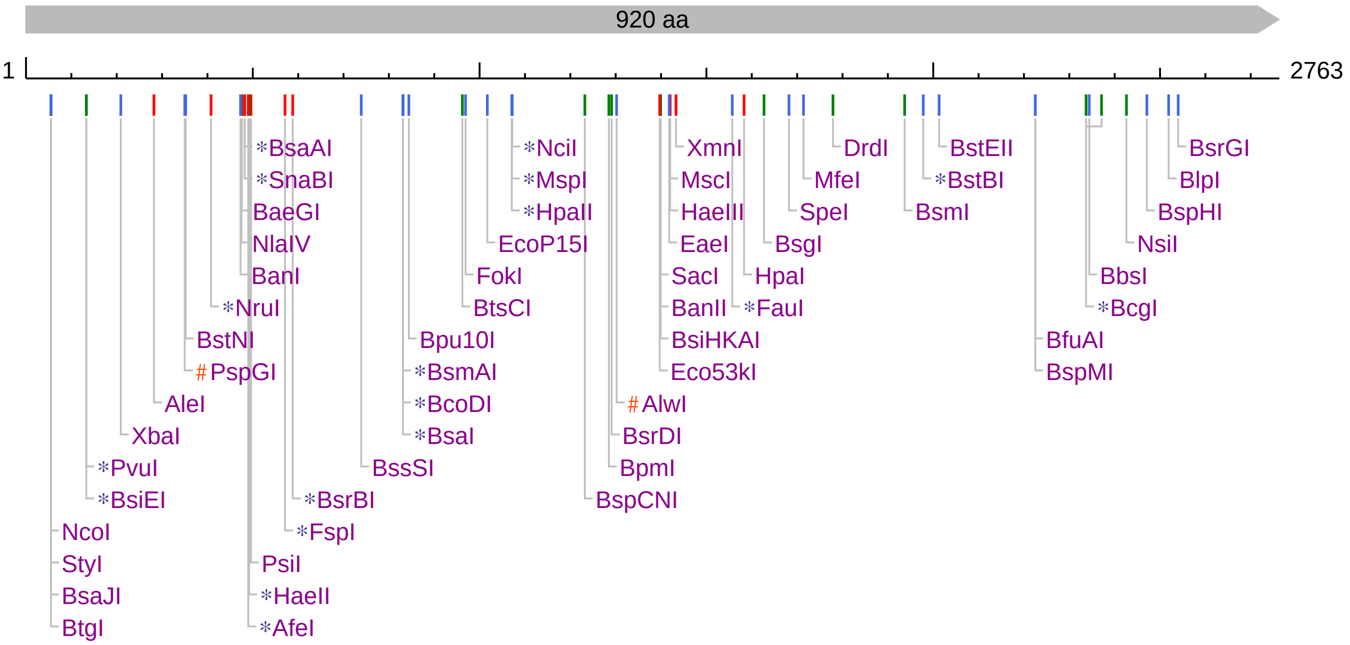

Supplement: Supplementary file 3 — Supplementary material [file mmc3.zip › AR360583 BioLab NEBcutter result.pdf]
