# Supplementary material for: Genomics dataset of unidentified disclosed isolates
Source: Data Brief. 2016 Jun 15;8:579–87. doi: 10.1016/j.dib.2016.06.010 (PMC4930343; doi:10.1016/j.dib.2016.06.010)
Supplement: Supplementary file 3 — Supplementary material [file mmc3.zip › AR360585 BioLab NEBcutter result.pdf]

NEW ENGLAND BioLabs Inc.
NEBcutter

Linear Sequence: AR360585

Display: - NEB restriction enzymes

GC=27%, AT=73%

Cleavage code	Enzyme name code
blunt end cut	Available from NEB
5’ extension	Has other supplier
3’ extension	Not commercially available
cuts 1 strand	*: cleavage affected by CpG methylation
	#: cleavage affected by other methylation
	(enz. name): ambiguous site

*MspJI MluCI MspJI MluCI *MspJI FspEI

5’ ... GTGAAAAAATTATTATTCGCAATTCCTTTAGTTGTTCCTTTCTAT ... 3’

1 10 20 30 40

3’ ... CACTTTTTTAATAATAAGCGTTAAGGAAATCAACAAGGAAAGATA ... 5’

*MspJI MluCI MspJI MluCI *MspJI FspEI
