# Supplementary material for: Genomics dataset of unidentified disclosed isolates
Source: Data Brief. 2016 Jun 15;8:579–87. doi: 10.1016/j.dib.2016.06.010 (PMC4930343; doi:10.1016/j.dib.2016.06.010)

NEBcutter

Linear Sequence: AX000222

Display: - NEB single cutter restriction enzymes
- Main non-overlapping, min. 100 aa ORFs

GC=36%, AT=64%

Cleavage code	Enzyme name code
blunt end cut	Available from NEB
5' extension	Has other supplier
3' extension	Not commercially available
cuts 1 strand	*: cleavage affected by CpG methylation
	#: cleavage affected by other methylation
	(enz. name): ambiguous site

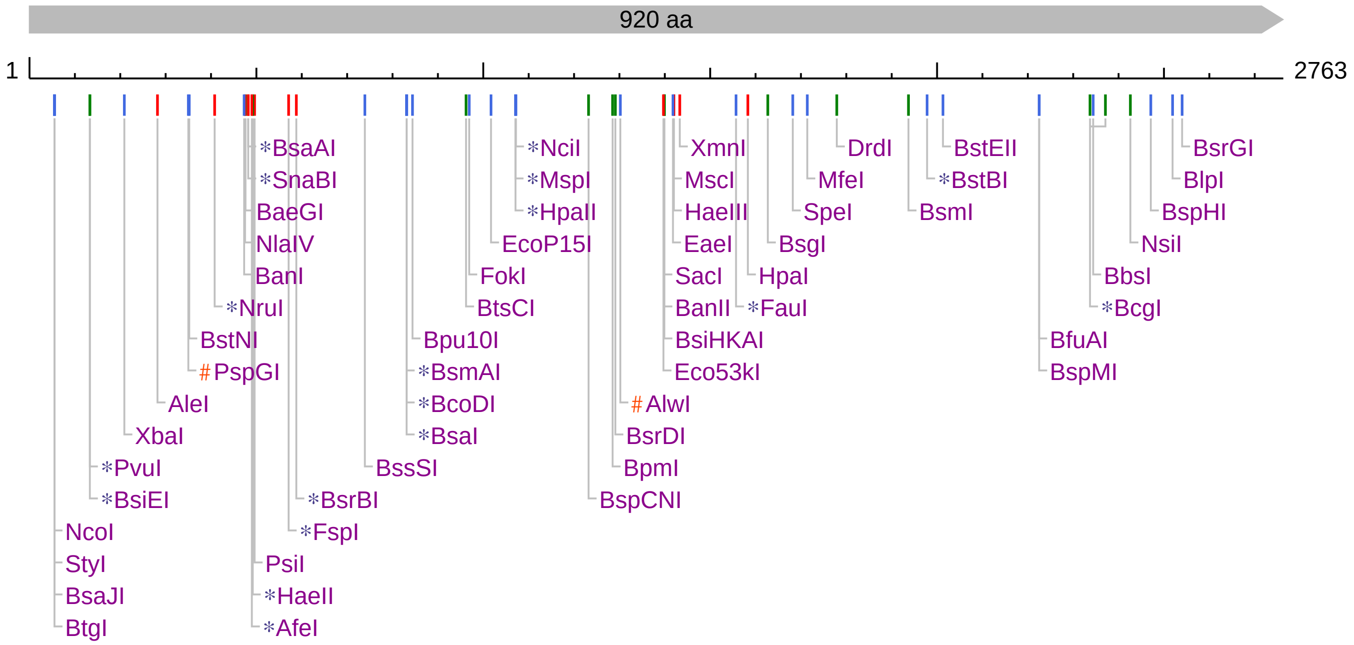

Supplement: Supplementary file 3 — Supplementary material [file mmc3.zip › AX000222 BioLab NEBcutter result.pdf]
