# Supplementary material for: Genomics dataset of unidentified disclosed isolates
Source: Data Brief. 2016 Jun 15;8:579–87. doi: 10.1016/j.dib.2016.06.010 (PMC4930343; doi:10.1016/j.dib.2016.06.010)
Supplement: Supplementary file 4 — Supplementary material [file mmc4.docx]

>gi|7240684|emb|AX000220.1| Sequence 3 from Patent WO9906567

TTAATCGATTCTAGATGGATAGGAAAAAAGCTG

>gi|7240685|emb|AX000221.1| Sequence 4 from Patent WO9906567

ATACCCGGGGGTACGGATCCGATACAGATTTGAGCAA

>gi|7240688|emb|AX000224.1| Sequence 7 from Patent WO9906567

ATGAAAATAAAAACAGGTGCACGCATCCTCGCATTATCCGCATTAACGACGATGATGTTTTCCGCCTCGG

CTCTC

>gi|7240689|emb|AX000225.1| Sequence 8 from Patent WO9906567

GTGAAAAAATTATTATTCGCAATTCCTTTAGTTGTTCCTTTCTAT

>gi|7240682|emb|AX000218.1| Sequence 1 from Patent WO9906567

ATGGATAGGAAAAAAGCTGTGAAACTAGCAACAGCAAGTGCTATTGCAGCAAGTGCATTTGTCGCTGCAA

ATCCAAACGCTTCTGAAGCGGCTACAGATGTAGCAACAGTAGTAAGCCAAGCAAAAGCACAGTTCAAAAA

AGCATACTATACTTACAGCCATACAGTAACGGAAACTGGTGAATTCCCAAACATTAACGATGTATATGCT

GAATACAACAAAGCGAAAAAACGATACCGTGATGCGGTAGCATTAGTGAATAAAGCAGGTGGCGCGAAAA

AAGACGCTTACTTAGCTGATTTACAAAAAGAATATGAAACTTACGTTTTCAAAGCAAACCCTAAATCTGG

CGAAGCTCGTGTAGCAACTTACATCGATGCTTACAACTATGCAACAAAATTAGACGAAATGCGCCAAGAG

CTAGAGGCTGCTGTTCAAGCAAAAGATTTAGAAAAAGCAGAACAATACTATCACAAAATTCCTTATGAAA

TTAAAACTCGCACAGTCATTTTAGATCGCGTATATGGTAAAACAACTCGTGATTTACTTCGCTCTACATT

TAAAGCAAAAGCACAAGAACTTCGCGACAGCTTAATTTATGATATTACCGTTGCAATGAAAGCGCGCGAA

GTACAAGACGCTGTGAAAGCAGGCAATTTAGACAAAGCTAAAGCTGCTGTTGATCAAATCAATCAATACT

TACCAAAAGTAACAGATGCTTTCAAAACTGAACTAACAGAAGTAGCGAAAAAAGCATTAGATGCAGATGA

AGCTGCGCTTACTCCAAAAGTTGAAAGTGTAAGTGCGATTAACACTCAAAACAAAGCTGTTGAATTAACA

GCAGTACCAGTGAACGGAACACTAAAATTACAACTTTCAGCTGCTGCAAATGAAGATACAGTAAACGTAA

ATACTGTACGTATCTATAAAGTGGACGGTAACATTCCATTTGCCCTTAATACGGCAGATGTTTCTTTATC

TACAGACGGAAAAACTATCACTGTGGATGCTTCAACTCCATTCGAAAATAATACGGAGTATAAAGTAGTA

GTTAAAGGTATTAAAGACAAAAATGGCAAAGAATTTAAAGAAGATGCATTCACTTTCAAGCTTCGAAATG

ATGCTGTAGTTACTCAAGTGTTTGGAACTAATGTAACAAACAACACTTCTGTAAACTTAGCAGCAGGTAC

TTTCGACACTGACGATACTTTAACAGTAGTATTTGATAAGTTGTTAGCACCTGAAACTGTAAACAGCTCG

AACGTTACTATTACAGATGTTGAAACTGGAAAACGCATTCCAGTAATTGCATCTACTTCTGGTTCTACAA

TTACTATTACGTTAAAAGAAGCGTTAGTAACTGGTAAACAATATAAACTTGCTATCAATAATGTTAAAAC

ATTAACTGGTTACAATGCAGAAGCTTACGAGTTAGTGTTCACTGCAAACGCATCAGCACCAACTGTTGCT

ACCGCTCCTACTACTTTAGGTGGTACAACTTTATCTACTGGTTCTCTTACAACAAATGTTTGGGGTAAAT

TGGCTGGTGGTGTGAATGAAGCTGGAACTTATTATCCTGGTCTTCAATTCACAACAACGTTTGCTACTAA

GTTAGACGAATCTACTTTAGCTGATAACTTTGTATTAGTTGAAAAAGAATCTGGTACAGTTGTTGCTTCT

GAACTAAAATATAATGCAGACGCTAAAATGGTAACTTTAGTGCCAAAAGCGGACCTTAAAGAAAATACAA

TCTATCAAATCAAAATTAAAAAAGGCTTGAAGTCCGATAAAGGTATTGAATTAGGCACTGTTAACGAGAA

AACATATGAGTTCAAAACTCAAGACTTAACTGCTCCTACAGTTATTAGCGTAACGTCTAAAAATGGCGAC

GCTGGATTAAAAGTAACTGAAGCTCAAGAATTTACTGTGAAGTTCTCAGAGAATTTAAATACATTTAATG

CTACAACCGTTTCGGGTAGCACAATCACATACGGTCAAGTTGCTGTAGTAAAAGCGGGTGCAAACTTATC

TGCTCTTACAGCAAGTGACATCATTCCAGCTAGTGTTGAAGCGGTTACTGGTCAAGATGGAACATACAAA

GTGAAAGTTGCTGCTAACCAATTAGAACGTAACCAAGGGTACAAATTAGTAGTGTTCGGTAAAGGTGCAA

CAGCTCCTGTTAAAGATGCTGCAAATGCAAATACTTTAGCAACTAACTATATCTATACATTTACAACTGA

AGGTCAAGACGTAACAGCACCAACGGTTACAAAAGTATTCAAAGGTGATTCTTTAAAAGACGCTGATGCA

GTTACTACACTTACGAACGTTGATGCAGGTCAAAAATTCACTATCCAATTTAGCGAAGAATTAAAAACTT

CTAGTGGTTCTTTAGTGGGTGGCAAAGTAACTGTCGAGAAATTAACAAACAACGGATGGGTAGATGCTGG

TACTGGAACAACTGTATCAGTTGCTCCTAAGACAGATGCAAATGGTAAAGTAACAGCTGCTGTGGTTACA

TTAACTGGTCTTGACAATAACGACAAAGATGCGAAATTGCGTCTGGTAGTAGATAAGTCTTCTACTGATG

GAATTGCTGATGTAGCTGGTAATGTAATTAAGGAAAAAGATATTTTAATTCGTTACAACAGCTGGAGACA

CACTGTAGCTTCTGTGAAAGCTGCTGCTGACAAAGATGGTCAAAACGCTTCTGCTGCATTCCCAACAAGC

ACTGCAATTGATACAACTAAGAGCTTATTAGTTGAATTCAATGAAACTGATTTAGCGGAAGTTAAACCTG

AGAACATCGTTGTTAAAGATGCAGCAGGTAATGCGGTAGCTGGTACTGTAACAGCATTAGACGGTTCTAC

AAATAAATTTGTATTCACTCCATCTCAAGAATTAAAAGCTGGTACAGTTTACTCTGTAACAATTGACGGT

GTGAGAGATAAAGTAGGTAACACAATCTCTAAATACATTACTTCGTTCAAGACTGTATCTGCGAATCCAA

CGTTATCTTCAATCAGCATTGCTGACGGTGCAGTTAACGTTGACCGTTCTAAAACAATTACAATTGAATT

CAGCGATTCAGTTCCAAACCCAACAATCACTCTTAAGAAGGCTGACGGAACTTCATTTACTAATTACACT

TTAGTAAATGTAAATAATGAAAATAAAACATACAAAATTGTATTCCACAAAGGTGTAACACTTGACGAGT

TTACTCAATATGAGTTAGCAGTTTCAAAAGATTTTCAAACTGGTACTGATATTGATAGCAAAGTTACATT

CATCACAGGTTCTGTTGCTACTGACGAAGTAAAACCTGCTCTAGTAGGCGTTGGTTCATGGAATGGAACA

AGCTATACTCAGGATGCTGCAGCAACACGACTTCGGTCTGTAGCTGACTTCGTTGCGGAGCCAGTTGCCC

TTCAATTCTCAGAAGGTATCGATTTAACGAATGCAACTGTGACAGTAACAAATATTACTGATGATAAAAC

TGTTGAAGTTATTTCAAAAGAGAGTGTAGACGCAGACCATGATGCAGGTGCTACTAAGGAGACATTAGTA

ATTAACACAGTTACTCCTTTAGTACTTGATAACAGCAAGACTTATAAGATTGTTGTAAGTGGAGTTAAAG

ATGCAGCAGGTAATGTTGCAGATACTATTACATTCTATATTAAGTAA

>gi|7240686|emb|AX000222.1| Sequence 5 from Patent WO9906567

ATGGCTTATCAACCTAAGTCCTATCGCAAGTTTGTTGCGACAACTGCAACAGCTGCCATGGTAGCATCTG

CGGTAGCTCCTGTAGTATCTGCAGCAAGCTTCACAGATGTTGCGCCGCAATATAAAGATGCGATCGATTT

CTTAGTATCAACTGGTGCAACAAAAGGTAAAACAGAAACAAAATTCGGCGTTTACGATGAAATCACTCGT

CTAGATGCGGCAGTTATTCTTGCAAGAGTATTAAAACTAGACGTTGACAACGCAAAAGACGCAGGCTTCA

CAGATGTGCCAAAAGACCGTGCAAAATACGTCAACGCGCTTGTAGAAGCTGGCGTATTAAACGGTAAAGC

ACCTGGCAAATTTGGTGCATACGACCCATTAACTCGCGTTGAAATGGCAAAAATCATCGCGAACCGTTAC

AAATTAAAAGCTGACGATGTAAAACTTCCATTCACTGATGTAAACGATACATGGGCACCATACGTAAAAG

CGCTTTATAAATACGAAGTAACAAAAGGTAAAACACCAACAAGCTTCGGTGCATACCAAAACATCACTCG

CGGTGACTTTGCGCAATTTGTATATAGAGCGGTGAATATTAATGCAGTGCCAGAAATAGTTGAAGTAACT

GCGGTTAATTCGACTACAGTGAAAGTAACATTCAATACGCAAATTGCTGATGTTGATTTCACAAATTTTG

CTATCGATAACGGTTTAACTGTTACTAAAGCAACTCTTTCTCGTGATAAAAAATCCGTAGAGGTTGTGGT

AAATAAACCGTTTACTCGTAATCAGGAATATACAATTACAGCGACAGGCATTAAAAATTTAAAAGGCGAG

ACCGCTAAGGAATTAACTGGTAAGTTTGTTTGGTCTGTTCAAGATGCGGTAACTGTTGCACTAAATAATA

GTTCGCTTAAAGTTGGAGAGGAATCTGGTTTAACTGTAAAAGATCAGGATGGCAAAGATGTTGTAGGTGC

TAAAGTAGAACTTACTTCTTCTAATACTAATATTGTTGTAGTTTCAAGTGGCGAAGTATCAGTATCTGCT

GCTAAAGTTACAGCTGTAAAACCGGGAACAGCTGATGTTACTGCAAAAGTTACATTACCAGATGGTGTTG

TACTAACAAATACATTTAAAGTGACAGTTACAGAAGTGCCTGTGCAAGTACAAAATCAAGGATTTACTTT

AGTTGATAATCTTTCTAATGCTCCACAGAATACAGTTGCATTTAACAAAGCTGAGAAAGTAACTTCAATG

TTTGCTGGAGAAACTAAAACAGTTGCAATGTATGATACTAAAAACGGTGATCCTGAAACTAAACCTGTTG

ATTTCAAAGATGCAACTGTACGTTCATTAAATCCAATTATTGCAACAGCTGCTATTAATGGTAGTGAGCT

CCTTGTCACAGCTAATGCTGGCCAATCTGGAAAAGCTTCATTTGAAGTAACATTTAAAGATAATACAAAA

AGAACATTTACAGTTGATGTGAAAAAAGACCCTGTATTACAAGATATTAAAGTAGATGCAACTTCTGTTA

AACTTTCCGATGAAGCTGTTGGCGGCGGGGAAGTTGAAGGAGTTAACCAAAAAACGATTAAAGTAAGTGC

AGTTGACCAATACGGTAAAGAAATTAAATTTGGTACAAAAGGTAAAGTTACTGTTACAACTAATACAGAA

GGACTAGTTATTAAAAATGTAAATAGCGATAATACAATTGACTTTGATAGCGGCAATAGTGCAACTGACC

AATTTGTTGTCGTTGCAACAAAAGACAAAATTGTCAATGGTAAAGTAGAAGTTAAATATTTCAAAAATGC

TAGTGACACAACACCAACTTCAACTAAAACAATTACTGTTAATGTAGTGAATGTAAAAGCTGACGCTACA

CCAGTAGGATTAGATATTGTAGCACCTTCTGAAATTGATGTGAATGCTCCAAACACTGCTTCTACTGCAG

ATGTTGATTTTATTAATTTCGAAAGTGTTGAGATTTATACACTCGATTCTAATGGTAACCGTCTTAAAAA

AGTTACTCCAACTGCAACTACACTTGTAGGTACTAATGATTATGTTGAAGTTAATGGGAATGTATTACAA

TTCAAGGGTAACGATGAATTAACGCTATTAACTTCTTCTAGTACAGTAAACGTTGATGTAACAGCTGATG

GAATTACAAAACGTATTCCAGTAAAATATATCAACTCTGCAAGTGTACCTGCCAGTGCAACAGTAGCAAC

AAGTCCTGTTACTGTTAAGCTTAATTCAAGTGATAATGATTTAACATTTGAAGAATTAATATTCGGTGTA

ATTGACCCTACACAATTAGTCAAAGATGAAGACATCAACGAATTTATTGCAGTTTCAAAAGCGGCTAAAA

ATGATGGATATTTGTATAATAAACCGCTTGTAACGGTTAAAGATGCATCAGGAAAAGTTATTCCAACAGG

TGCAAATGTTTACGGTCTAAATCATGATGCAACTAACGGAAACATTTGGTTTGATGAGGAACAAGCTGGC

TTAGCTAAAAAATTTAGTGATGTACATTTTGATGTTGATTTTTCATTAGCTAACGTTGTAAAAACTGGTA

GCGGTACAGTTTCTTCATCGCCATCATTATCTGACGCAATTCAACTTACTAATTCAGGCGATGCAGTATC

GTTTACATTAGTTATCAAATCAATTTATGTTAAAGGCGCAGATAAAGATGATAATAACTTACTTGCAGCC

CCTGTTTCTGTCAATGTGACTGTGACAAAATAA

>gi|33767646|gb|AR360580.1| Sequence 1 from patent US 6596510

ATGGATAGGAAAAAAGCTGTGAAACTAGCAACAGCAAGTGCTATTGCAGCAAGTGCATTTGTCGCTGCAA

ATCCAAACGCTTCTGAAGCGGCTACAGATGTAGCAACAGTAGTAAGCCAAGCAAAAGCACAGTTCAAAAA

AGCATACTATACTTACAGCCATACAGTAACGGAAACTGGTGAATTCCCAAACATTAACGATGTATATGCT

GAATACAACAAAGCGAAAAAACGATACCGTGATGCGGTAGCATTAGTGAATAAAGCAGGTGGCGCGAAAA

AAGACGCTTACTTAGCTGATTTACAAAAAGAATATGAAACTTACGTTTTCAAAGCAAACCCTAAATCTGG

CGAAGCTCGTGTAGCAACTTACATCGATGCTTACAACTATGCAACAAAATTAGACGAAATGCGCCAAGAG

CTAGAGGCTGCTGTTCAAGCAAAAGATTTAGAAAAAGCAGAACAATACTATCACAAAATTCCTTATGAAA

TTAAAACTCGCACAGTCATTTTAGATCGCGTATATGGTAAAACAACTCGTGATTTACTTCGCTCTACATT

TAAAGCAAAAGCACAAGAACTTCGCGACAGCTTAATTTATGATATTACCGTTGCAATGAAAGCGCGCGAA

GTACAAGACGCTGTGAAAGCAGGCAATTTAGACAAAGCTAAAGCTGCTGTTGATCAAATCAATCAATACT

TACCAAAAGTAACAGATGCTTTCAAAACTGAACTAACAGAAGTAGCGAAAAAAGCATTAGATGCAGATGA

AGCTGCGCTTACTCCAAAAGTTGAAAGTGTAAGTGCGATTAACACTCAAAACAAAGCTGTTGAATTAACA

GCAGTACCAGTGAACGGAACACTAAAATTACAACTTTCAGCTGCTGCAAATGAAGATACAGTAAACGTAA

ATACTGTACGTATCTATAAAGTGGACGGTAACATTCCATTTGCCCTTAATACGGCAGATGTTTCTTTATC

TACAGACGGAAAAACTATCACTGTGGATGCTTCAACTCCATTCGAAAATAATACGGAGTATAAAGTAGTA

GTTAAAGGTATTAAAGACAAAAATGGCAAAGAATTTAAAGAAGATGCATTCACTTTCAAGCTTCGAAATG

ATGCTGTAGTTACTCAAGTGTTTGGAACTAATGTAACAAACAACACTTCTGTAAACTTAGCAGCAGGTAC

TTTCGACACTGACGATACTTTAACAGTAGTATTTGATAAGTTGTTAGCACCTGAAACTGTAAACAGCTCG

AACGTTACTATTACAGATGTTGAAACTGGAAAACGCATTCCAGTAATTGCATCTACTTCTGGTTCTACAA

TTACTATTACGTTAAAAGAAGCGTTAGTAACTGGTAAACAATATAAACTTGCTATCAATAATGTTAAAAC

ATTAACTGGTTACAATGCAGAAGCTTACGAGTTAGTGTTCACTGCAAACGCATCAGCACCAACTGTTGCT

ACCGCTCCTACTACTTTAGGTGGTACAACTTTATCTACTGGTTCTCTTACAACAAATGTTTGGGGTAAAT

TGGCTGGTGGTGTGAATGAAGCTGGAACTTATTATCCTGGTCTTCAATTCACAACAACGTTTGCTACTAA

GTTAGACGAATCTACTTTAGCTGATAACTTTGTATTAGTTGAAAAAGAATCTGGTACAGTTGTTGCTTCT

GAACTAAAATATAATGCAGACGCTAAAATGGTAACTTTAGTGCCAAAAGCGGACCTTAAAGAAAATACAA

TCTATCAAATCAAAATTAAAAAAGGCTTGAAGTCCGATAAAGGTATTGAATTAGGCACTGTTAACGAGAA

AACATATGAGTTCAAAACTCAAGACTTAACTGCTCCTACAGTTATTAGCGTAACGTCTAAAAATGGCGAC

GCTGGATTAAAAGTAACTGAAGCTCAAGAATTTACTGTGAAGTTCTCAGAGAATTTAAATACATTTAATG

CTACAACCGTTTCGGGTAGCACAATCACATACGGTCAAGTTGCTGTAGTAAAAGCGGGTGCAAACTTATC

TGCTCTTACAGCAAGTGACATCATTCCAGCTAGTGTTGAAGCGGTTACTGGTCAAGATGGAACATACAAA

GTGAAAGTTGCTGCTAACCAATTAGAACGTAACCAAGGGTACAAATTAGTAGTGTTCGGTAAAGGTGCAA

CAGCTCCTGTTAAAGATGCTGCAAATGCAAATACTTTAGCAACTAACTATATCTATACATTTACAACTGA

AGGTCAAGACGTAACAGCACCAACGGTTACAAAAGTATTCAAAGGTGATTCTTTAAAAGACGCTGATGCA

GTTACTACACTTACGAACGTTGATGCAGGTCAAAAATTCACTATCCAATTTAGCGAAGAATTAAAAACTT

CTAGTGGTTCTTTAGTGGGTGGCAAAGTAACTGTCGAGAAATTAACAAACAACGGATGGGTAGATGCTGG

TACTGGAACAACTGTATCAGTTGCTCCTAAGACAGATGCAAATGGTAAAGTAACAGCTGCTGTGGTTACA

TTAACTGGTCTTGACAATAACGACAAAGATGCGAAATTGCGTCTGGTAGTAGATAAGTCTTCTACTGATG

GAATTGCTGATGTAGCTGGTAATGTAATTAAGGAAAAAGATATTTTAATTCGTTACAACAGCTGGAGACA

CACTGTAGCTTCTGTGAAAGCTGCTGCTGACAAAGATGGTCAAAACGCTTCTGCTGCATTCCCAACAAGC

ACTGCAATTGATACAACTAAGAGCTTATTAGTTGAATTCAATGAAACTGATTTAGCGGAAGTTAAACCTG

AGAACATCGTTGTTAAAGATGCAGCAGGTAATGCGGTAGCTGGTACTGTAACAGCATTAGACGGTTCTAC

AAATAAATTTGTATTCACTCCATCTCAAGAATTAAAAGCTGGTACAGTTTACTCTGTAACAATTGACGGT

GTGAGAGATAAAGTAGGTAACACAATCTCTAAATACATTACTTCGTTCAAGACTGTATCTGCGAATCCAA

CGTTATCTTCAATCAGCATTGCTGACGGTGCAGTTAACGTTGACCGTTCTAAAACAATTACAATTGAATT

CAGCGATTCAGTTCCAAACCCAACAATCACTCTTAAGAAGGCTGACGGAACTTCATTTACTAATTACACT

TTAGTAAATGTAAATAATGAAAATAAAACATACAAAATTGTATTCCACAAAGGTGTAACACTTGACGAGT

TTACTCAATATGAGTTAGCAGTTTCAAAAGATTTTCAAACTGGTACTGATATTGATAGCAAAGTTACATT

CATCACAGGTTCTGTTGCTACTGACGAAGTAAAACCTGCTCTAGTAGGCGTTGGTTCATGGAATGGAACA

AGCTATACTCAGGATGCTGCAGCAACACGACTTCGGTCTGTAGCTGACTTCGTTGCGGAGCCAGTTGCCC

TTCAATTCTCAGAAGGTATCGATTTAACGAATGCAACTGTGACAGTAACAAATATTACTGATGATAAAAC

TGTTGAAGTTATTTCAAAAGAGAGTGTAGACGCAGACCATGATGCAGGTGCTACTAAGGAGACATTAGTA

ATTAACACAGTTACTCCTTTAGTACTTGATAACAGCAAGACTTATAAGATTGTTGTAAGTGGAGTTAAAG

ATGCAGCAGGTAATGTTGCAGATACTATTACATTCTATATTAAGTAA

>gi|33767647|gb|AR360581.1| Sequence 3 from patent US 6596510

TTAATCGATTCTAGATGGATAGGAAAAAAGCTG

>gi|33767648|gb|AR360582.1| Sequence 4 from patent US 6596510

ATACCCGGGGGTACGGATCCGATACAGATTTGAGCAA

>gi|33767649|gb|AR360583.1| Sequence 5 from patent US 6596510

ATGGCTTATCAACCTAAGTCCTATCGCAAGTTTGTTGCGACAACTGCAACAGCTGCCATGGTAGCATCTG

CGGTAGCTCCTGTAGTATCTGCAGCAAGCTTCACAGATGTTGCGCCGCAATATAAAGATGCGATCGATTT

CTTAGTATCAACTGGTGCAACAAAAGGTAAAACAGAAACAAAATTCGGCGTTTACGATGAAATCACTCGT

CTAGATGCGGCAGTTATTCTTGCAAGAGTATTAAAACTAGACGTTGACAACGCAAAAGACGCAGGCTTCA

CAGATGTGCCAAAAGACCGTGCAAAATACGTCAACGCGCTTGTAGAAGCTGGCGTATTAAACGGTAAAGC

ACCTGGCAAATTTGGTGCATACGACCCATTAACTCGCGTTGAAATGGCAAAAATCATCGCGAACCGTTAC

AAATTAAAAGCTGACGATGTAAAACTTCCATTCACTGATGTAAACGATACATGGGCACCATACGTAAAAG

CGCTTTATAAATACGAAGTAACAAAAGGTAAAACACCAACAAGCTTCGGTGCATACCAAAACATCACTCG

CGGTGACTTTGCGCAATTTGTATATAGAGCGGTGAATATTAATGCAGTGCCAGAAATAGTTGAAGTAACT

GCGGTTAATTCGACTACAGTGAAAGTAACATTCAATACGCAAATTGCTGATGTTGATTTCACAAATTTTG

CTATCGATAACGGTTTAACTGTTACTAAAGCAACTCTTTCTCGTGATAAAAAATCCGTAGAGGTTGTGGT

AAATAAACCGTTTACTCGTAATCAGGAATATACAATTACAGCGACAGGCATTAAAAATTTAAAAGGCGAG

ACCGCTAAGGAATTAACTGGTAAGTTTGTTTGGTCTGTTCAAGATGCGGTAACTGTTGCACTAAATAATA

GTTCGCTTAAAGTTGGAGAGGAATCTGGTTTAACTGTAAAAGATCAGGATGGCAAAGATGTTGTAGGTGC

TAAAGTAGAACTTACTTCTTCTAATACTAATATTGTTGTAGTTTCAAGTGGCGAAGTATCAGTATCTGCT

GCTAAAGTTACAGCTGTAAAACCGGGAACAGCTGATGTTACTGCAAAAGTTACATTACCAGATGGTGTTG

TACTAACAAATACATTTAAAGTGACAGTTACAGAAGTGCCTGTGCAAGTACAAAATCAAGGATTTACTTT

AGTTGATAATCTTTCTAATGCTCCACAGAATACAGTTGCATTTAACAAAGCTGAGAAAGTAACTTCAATG

TTTGCTGGAGAAACTAAAACAGTTGCAATGTATGATACTAAAAACGGTGATCCTGAAACTAAACCTGTTG

ATTTCAAAGATGCAACTGTACGTTCATTAAATCCAATTATTGCAACAGCTGCTATTAATGGTAGTGAGCT

CCTTGTCACAGCTAATGCTGGCCAATCTGGAAAAGCTTCATTTGAAGTAACATTTAAAGATAATACAAAA

AGAACATTTACAGTTGATGTGAAAAAAGACCCTGTATTACAAGATATTAAAGTAGATGCAACTTCTGTTA

AACTTTCCGATGAAGCTGTTGGCGGCGGGGAAGTTGAAGGAGTTAACCAAAAAACGATTAAAGTAAGTGC

AGTTGACCAATACGGTAAAGAAATTAAATTTGGTACAAAAGGTAAAGTTACTGTTACAACTAATACAGAA

GGACTAGTTATTAAAAATGTAAATAGCGATAATACAATTGACTTTGATAGCGGCAATAGTGCAACTGACC

AATTTGTTGTCGTTGCAACAAAAGACAAAATTGTCAATGGTAAAGTAGAAGTTAAATATTTCAAAAATGC

TAGTGACACAACACCAACTTCAACTAAAACAATTACTGTTAATGTAGTGAATGTAAAAGCTGACGCTACA

CCAGTAGGATTAGATATTGTAGCACCTTCTGAAATTGATGTGAATGCTCCAAACACTGCTTCTACTGCAG

ATGTTGATTTTATTAATTTCGAAAGTGTTGAGATTTATACACTCGATTCTAATGGTAACCGTCTTAAAAA

AGTTACTCCAACTGCAACTACACTTGTAGGTACTAATGATTATGTTGAAGTTAATGGGAATGTATTACAA

TTCAAGGGTAACGATGAATTAACGCTATTAACTTCTTCTAGTACAGTAAACGTTGATGTAACAGCTGATG

GAATTACAAAACGTATTCCAGTAAAATATATCAACTCTGCAAGTGTACCTGCCAGTGCAACAGTAGCAAC

AAGTCCTGTTACTGTTAAGCTTAATTCAAGTGATAATGATTTAACATTTGAAGAATTAATATTCGGTGTA

ATTGACCCTACACAATTAGTCAAAGATGAAGACATCAACGAATTTATTGCAGTTTCAAAAGCGGCTAAAA

ATGATGGATATTTGTATAATAAACCGCTTGTAACGGTTAAAGATGCATCAGGAAAAGTTATTCCAACAGG

TGCAAATGTTTACGGTCTAAATCATGATGCAACTAACGGAAACATTTGGTTTGATGAGGAACAAGCTGGC

TTAGCTAAAAAATTTAGTGATGTACATTTTGATGTTGATTTTTCATTAGCTAACGTTGTAAAAACTGGTA

GCGGTACAGTTTCTTCATCGCCATCATTATCTGACGCAATTCAACTTACTAATTCAGGCGATGCAGTATC

GTTTACATTAGTTATCAAATCAATTTATGTTAAAGGCGCAGATAAAGATGATAATAACTTACTTGCAGCC

CCTGTTTCTGTCAATGTGACTGTGACAAAATAA

>gi|33767650|gb|AR360584.1| Sequence 7 from patent US 6596510

ATGAAAATAAAAACAGGTGCACGCATCCTCGCATTATCCGCATTAACGACGATGATGTTTTCCGCCTCGG

CTCTC

>gi|33767651|gb|AR360585.1| Sequence 8 from patent US 6596510

GTGAAAAAATTATTATTCGCAATTCCTTTAGTTGTTCCTTTCTAT

>gi|33767652|gb|AR360586.1| Sequence 9 from patent US 6596510

GAATTCATCGATGTCGACCAAGGAGGTCTAGATGGATCCGGCCAAGCTT

>gi|33767653|gb|AR360587.1| Sequence 10 from patent US 6596510

ATCGAGGGAAGGATTTCAGAATTCGGATCCTCTAGAGTCGACCTGCAGGCAAGCTTG

>gi|33767654|gb|AR360588.1| Sequence 11 from patent US 6596510

ATGAAAATAAAAACAGGTGCACGCATCCTCGCATTATCCGCATTAACGACGATGATGTTTTCCGCCTCGG

CTCTCGCCAAAATCGAAGAAGGTAAACTGGTAATCTGGATTAACGGCGATAAAGGCTATAACGGTCTCGC

TGAAGTCGGTAAGAAATTCGAGAAAGATACCGGAATTAAAGTCACCGTTGAGCATCCGGATAAACTGGAA

GAGAAATTCCCACAGGTTGCGGCAACTGGCGATGGCCCTGACATTATCTTCTGGGCACACGACCGCTTTG

GTGGCTACGCTCAATCTGGCCTGTTGGCTGAAATCACCCCGGACAAAGCGTTCCAGGACAAGCTGTATCC

GTTTACCTGGGATGCCGTACGTTACAACGGCAAGCTGATTGCTTACCCGATCGCTGTTGAAGCGTTATCG

CTGATTTATAACAAAGATCTGCTGCCGAACCCGCCAAAAACCTGGGAAGAGATCCCGGCGCTGGATAAAG

AACTGAAAGCGAAAGGTAAGAGCGCGCTGATGTTCAACCTGCAAGAACCGTACTTCACCTGGCCGCTGAT

TGCTGCTGACGGGGGTTATGCGTTCAAGTATGAAAACGGCAAGTACGACATTAAAGACGTGGGCGTGGAT

AACGCTGGCGCGAAAGCGGGTCTGACCTTCCTGGTTGACCTGATTAAAAACAAACACATGAATGCAGACA

CCGATTACTCCATCGCAGAAGCTGCCTTTAATAAAGGCGAAACAGCGATGACCATCAACGGCCCGTGGGC

ATGGTCCAACATCGACACCAGCAAATTGAATTATGGTGTAACGGTACTGCCGACCTTCAAGGGTCACCCA

TCCAAACCGTTCGTTGGCGTGCTGAGCGCAGGTATTAACGCCGCCAGTCCGAACAAAGAGTTGGCGAAAG

AGTTCCTCGAAAACTATCTGCTGACTGATGAAGGTCTGGAAGCGGTTAATAAAGACAAACCGCTGGGTGC

CGTAGCGCTGAAGTCTTACGAGGAAGAGTTGGCGAAAGATCCACGTATTGCCGCCACCATGGAAAACGCC

CAGAAAGGTGAAATCATGCCGAACATCCCGCAGATGTCCGCTTTCTGGTATGCCGTGCGTACTGCGGTGA

TCAACGCCGCCAGCGGTCGTCAGATCGTCGATGAAGCCCTGAAAGACGCGCAGACTAATTCGAGCTCGAA

CAACAACAACAATAACAATAACAACAACCTCGGGATCGAGGGAAGGATTTCAGAATTCGGATCCGCTACA

GATGTAGCAACAGTAGTAAGCCAAGCAAAAGCACAGTTCAAAAAAGCATACTATACTTACAGCCATACAG

TAACGGAAACTGGTGAATTCCCAAACATTAACGATGTATATGCTGAATACAACAAAGCGAAAAAACGATA

CCGTGATGCGGTAGCATTAGTGAATAAAGCAGGTGGCGCGAAAAAAGACGCTTACTTAGCTGATTTACAA

AAAGAATATGAAACTTACGTTTTCAAAGCAAACCCTAAATCTGGCGAAGCTCGTGTAGCAACTTACATCG

ATGCTTACAACTATGCAACAAAATTAGACGAAATGCGCCAAGAGCTAGAGGCTGCTGTTCAAGCAAAAGA

TTTAGAAAAAGCAGAACAATACTATCACAAAATTCCTTATGAAATTAAAACTCGCACAGTCATTTTAGAT

CGCGTATATGGTAAAACAACTCGTGATTTACTTCGCTCTACATTTAAAGCAAAAGCACAAGAACTTCGCG

ACAGCTTAATTTATGATATTACCGTTGCAATGAAAGCGCGCGAAGTACAAGACGCTGTGAAAGCAGGCAA

TTTAGACAAAGCTAAAGCTGCTGTTGATCAAATCAATCAATACTTACCAAAAGTAACAGATGCTTTCAAA

ACTGAACTAACAGAAGTAGCGAAAAAAGCATTAGATGCAGATGAAGCTGCGCTTACTCCAAAAGTTGAAA

GTGTAAGTGCGATTAACACTCAAAACAAAGCTGTTGAATTAACAGCAGTACCAGTGAACGGAACACTAAA

ATTACAACTTTCAGCTGCTGCAAATGAAGATACAGTAAACGTAAATACTGTACGTATCTATAAAGTGGAC

GGTAACATTCCATTTGCCCTTAATACGGCAGATGTTTCTTTATCTACAGACGGAAAAACTATCACTGTGG

ATGCTTCAACTCCATTCGAAAATAATACGGAGTATAAAGTAGTAGTTAAAGGTATTAAAGACAAAAATGG

CAAAGAATTTAAAGAAGATGCATTCACTTTCAAGCTTCGAAATGATGCTGTAGTTACTCAAGTGTTTGGA

ACTAATGTAACAAACAACACTTCTGTAAACTTAGCAGCAGGTACTTTCGACACTGACGATACTTTAACAG

TAGTATTTGATAAGTTGTTAGCACCTGAAACTGTAAACAGCTCGAACGTTACTATTACAGATGTTGAAAC

TGGAAAACGCATTCCAGTAATTGCATCTACTTCTGGTTCTACAATTACTATTACGTTAAAAGAAGCGTTA

GTAACTGGTAAACAATATAAACTTGCTATCAATAATGTTAAAACATTAACTGGTTACAATGCAGAAGCTT

ACGAGTTAGTGTTCACTGCAAACGCATCAGCACCAACTGTTGCTACCGCTCCTACTACTTTAGGTGGTAC

AACTTTATCTACTGGTTCTCTTACAACAAATGTTTGGGGTAAATTGGCTGGTGGTGTGAATGAAGCTGGA

ACTTATTATCCTGGTCTTCAATTCACAACAACGTTTGCTACTAAGTTAGACGAATCTACTTTAGCTGATA

ACTTTGTATTAGTTGAAAAAGAATCTGGTACAGTTGTTGCTTCTGAACTAAAATATAATGCAGACGCTAA

AATGGTAACTTTAGTGCCAAAAGCGGACCTTAAAGAAAATACAATCTATCAAATCAAAATTAAAAAAGGC

TTGAAGTCCGATAAAGGTATTGAATTAGGCACTGTTAACGAGAAAACATATGAGTTCAAAACTCAAGACT

TAACTGCTCCTACAGTTATTAGCGTAACGTCTAAAAATGGCGACGCTGGATTAAAAGTAACTGAAGCTCA

AGAATTTACTGTGAAGTTCTCAGAGAATTTAAATACATTTAATGCTACAACCGTTTCGGGTAGCACAATC

ACATACGGTCAAGTTGCTGTAGTAAAAGCGGGTGCAAACTTATCTGCTCTTACAGCAAGTGACATCATTC

CAGCTAGTGTTGAAGCGGTTACTGGTCAAGATGGAACATACAAAGTGAAAGTTGCTGCTAACCAATTAGA

ACGTAACCAAGGGTACAAATTAGTAGTGTTCGGTAAAGGTGCAACAGCTCCTGTTAAAGATGCTGCAAAT

GCAAATACTTTAGCAACTAACTATATCTATACATTTACAACTGAAGGTCAAGACGTAACAGCACCAACGG

TTACAAAAGTATTCAAAGGTGATTCTTTAAAAGACGCTGATGCAGTTACTACACTTACGAACGTTGATGC

AGGTCAAAAATTCACTATCCAATTTAGCGAAGAATTAAAAACTTCTAGTGGTTCTTTAGTGGGTGGCAAA

GTAACTGTCGAGAAATTAACAAACAACGGATGGGTAGATGCTGGTACTGGAACAACTGTATCAGTTGCTC

CTAAGACAGATGCAAATGGTAAAGTAACAGCTGCTGTGGTTACATTAACTGGTCTTGACAATAACGACAA

AGATGCGAAATTGCGTCTGGTAGTAGATAAGTCTTCTACTGATGGAATTGCTGATGTAGCTGGTAATGTA

ATTAAGGAAAAAGATATTTTAATTCGTTACAACAGCTGGAGACACACTGTAGCTTCTGTGAAAGCTGCTG

CTGACAAAGATGGTCAAAACGCTTCTGCTGCATTCCCAACAAGCACTGCAATTGATACAACTAAGAGCTT

ATTAGTTGAATTCAATGAAACTGATTTAGCGGAAGTTAAACCTGAGAACATCGTTGTTAAAGATGCAGCA

GGTAATGCGGTAGCTGGTACTGTAACAGCATTAGACGGTTCTACAAATAAATTTGTATTCACTCCATCTC

AAGAATTAAAAGCTGGTACAGTTTACTCTGTAACAATTGACGGTGTGAGAGATAAAGTAGGTAACACAAT

CTCTAAATACATTACTTCGTTCAAGACTGTATCTGCGAATCCAACGTTATCTTCAATCAGCATTGCTGAC

GGTGCAGTTAACGTTGACCGTTCTAAAACAATTACAATTGAATTCAGCGATTCAGTTCCAAACCCAACAA

TCACTCTTAAGAAGGCTGACGGAACTTCATTTACTAATTACACTTTAGTAAATGTAAATAATGAAAATAA

AACATACAAAATTGTATTCCACAAAGGTGTAACACTTGACGAGTTTACTCAATATGAGTTAGCAGTTTCA

AAAGATTTTCAAACTGGTACTGATATTGATAGCAAAGTTACATTCATCACAGGTTCTGTTGCTACTGACG

AAGTAAAACCTGCTCTAGTAGGCGTTGGTTCATGGAATGGAACAAGCTATACTCAGGATGCTGCAGCAAC

ACGACTTCGGTCTGTAGCTGACTTCGTTGCGGAGCCAGTTGCCCTTCAATTCTCAGAAGGTATCGATTTA

ACGAATGCAACTGTGACAGTAACAAATATTACTGATGATAAAACTGTTGAAGTTATTTCAAAAGAGAGTG

TAGACGCAGACCATGATGCAGGTGCTACTAAGGAGACATTAGTAATTAACACAGTTACTCCTTTAGTACT

TGATAACAGCAAGACTTATAAGATTGTTGTAAGTGGAGTTAAAGATGCAGCAGGTAATGTTGCAGATACT

ATTACATTCTATATTAAGTAATCTGGGCTAGGTGTTTGTCACCGCTCAAGGTTGTCAAAATATGTCGAAA

AGCTCTGCGGAGAGAAATCTCTGCGGGGCTTTTCTTTTTGCTCAAATCTGTATCAGGATCCTCTAGAGTC

GACCTGCAGGCAAGCTTG

>gi|33767655|gb|AR360589.1| Sequence 12 from patent US 6596510

GTGAAAAAATTATTATTCGCAATTCCTTTAGTTGTTCCTTTCTATGCGGCCCAGCCGGCCGCTACAGATG

TAGCAACAGTAGTAAGCCAAGCAAAAGCACAGTTCAAAAAAGCATACTATACTTACAGCCATACAGTAAC

GGAAACTGGTGAATTCCCAAACATTAACGATGTATATGCTGAATACAACAAAGCGAAAAAACGATACCGT

GATGCGGTAGCATTAGTGAATAAAGCAGGTGGCGCGAAAAAAGACGCTTACTTAGCTGATTTACAAAAAG

AATATGAAACTTACGTTTTCAAAGCAAACCCTAAATCTGGCGAAGCTCGTGTAGCAACTTACATCGATGC

TTACAACTATGCAACAAAATTAGACGAAATGCGCCAAGAGCTAGAGGCTGCTGTTCAAGCAAAAGATTTA

GAAAAAGCAGAACAATACTATCACAAAATTCCTTATGAAATTAAAACTCGCACAGTCATTTTAGATCGCG

TATATGGTAAAACAACTCGTGATTTACTTCGCTCTACATTTAAAGCAAAAGCACAAGAACTTCGCGACAG

CTTAATTTATGATATTACCGTTGCAATGAAAGCGCGCGAAGTACAAGACGCTGTGAAAGCAGGCAATTTA

GACAAAGCTAAAGCTGCTGTTGATCAAATCAATCAATACTTACCAAAAGTAACAGATGCTTTCAAAACTG

AACTAACAGAAGTAGCGAAAAAAGCATTAGATGCAGATGAAGCTGCGCTTACTCCAAAAGTTGAAAGTGT

AAGTGCGATTAACACTCAAAACAAAGCTGTTGAATTAACAGCAGTACCAGTGAACGGAACACTAAAATTA

CAACTTTCAGCTGCTGCAAATGAAGATACAGTAAACGTAAATACTGTACGTATCTATAAAGTGGACGGTA

ACATTCCATTTGCCCTTAATACGGCAGATGTTTCTTTATCTACAGACGGAAAAACTATCACTGTGGATGC

TTCAACTCCATTCGAAAATAATACGGAGTATAAAGTAGTAGTTAAAGGTATTAAAGACAAAAATGGCAAA

GAATTTAAAGAAGATGCATTCACTTTCAAGCTTCGAAATGATGCTGTAGTTACTCAAGTGTTTGGAACTA

ATGTAACAAACAACACTTCTGTAAACTTAGCAGCAGGTACTTTCGACACTGACGATACTTTAACAGTAGT

ATTTGATAAGTTGTTAGCACCTGAAACTGTAAACAGCTCGAACGTTACTATTACAGATGTTGAAACTGGA

AAACGCATTCCAGTAATTGCATCTACTTCTGGTTCTACAATTACTATTACGTTAAAAGAAGCGTTAGTAA

CTGGTAAACAATATAAACTTGCTATCAATAATGTTAAAACATTAACTGGTTACAATGCAGAAGCTTACGA

GTTAGTGTTCACTGCAAACGCATCAGCACCAACTGTTGCTACCGCTCCTACTACTTTAGGTGGTACAACT

TTATCTACTGGTTCTCTTACAACAAATGTTTGGGGTAAATTGGCTGGTGGTGTGAATGAAGCTGGAACTT

ATTATCCTGGTCTTCAATTCACAACAACGTTTGCTACTAAGTTAGACGAATCTACTTTAGCTGATAACTT

TGTATTAGTTGAAAAAGAATCTGGTACAGTTGTTGCTTCTGAACTAAAATATAATGCAGACGCTAAAATG

GTAACTTTAGTGCCAAAAGCGGACCTTAAAGAAAATACAATCTATCAAATCAAAATTAAAAAAGGCTTGA

AGTCCGATAAAGGTATTGAATTAGGCACTGTTAACGAGAAAACATATGAGTTCAAAACTCAAGACTTAAC

TGCTCCTACAGTTATTAGCGTAACGTCTAAAAATGGCGACGCTGGATTAAAAGTAACTGAAGCTCAAGAA

TTTACTGTGAAGTTCTCAGAGAATTTAAATACATTTAATGCTACAACCGTTTCGGGTAGCACAATCACAT

ACGGTCAAGTTGCTGTAGTAAAAGCGGGTGCAAACTTATCTGCTCTTACAGCAAGTGACATCATTCCAGC

TAGTGTTGAAGCGGTTACTGGTCAAGATGGAACATACAAAGTGAAAGTTGCTGCTAACCAATTAGAACGT

AACCAAGGGTACAAATTAGTAGTGTTCGGTAAAGGTGCAACAGCTCCTGTTAAAGATGCTGCAAATGCAA

ATACTTTAGCAACTAACTATATCTATACATTTACAACTGAAGGTCAAGACGTAACAGCACCAACGGTTAC

AAAAGTATTCAAAGGTGATTCTTTAAAAGACGCTGATGCAGTTACTACACTTACGAACGTTGATGCAGGT

CAAAAATTCACTATCCAATTTAGCGAAGAATTAAAAACTTCTAGTGGTTCTTTAGTGGGTGGCAAAGTAA

CTGTCGAGAAATTAACAAACAACGGATGGGTAGATGCTGGTACTGGAACAACTGTATCAGTTGCTCCTAA

GACAGATGCAAATGGTAAAGTAACAGCTGCTGTGGTTACATTAACTGGTCTTGACAATAACGACAAAGAT

GCGAAATTGCGTCTGGTAGTAGATAAGTCTTCTACTGATGGAATTGCTGATGTAGCTGGTAATGTAATTA

AGGAAAAAGATATTTTAATTCGTTACAACAGCTGGAGACACACTGTAGCTTCTGTGAAAGCTGCTGCTGA

CAAAGATGGTCAAAACGCTTCTGCTGCATTCCCAACAAGCACTGCAATTGATACAACTAAGAGCTTATTA

GTTGAATTCAATGAAACTGATTTAGCGGAAGTTAAACCTGAGAACATCGTTGTTAAAGATGCAGCAGGTA

ATGCGGTAGCTGGTACTGTAACAGCATTAGACGGTTCTACAAATAAATTTGTATTCACTCCATCTCAAGA

ATTAAAAGCTGGTACAGTTTACTCTGTAACAATTGACGGTGTGAGAGATAAAGTAGGTAACACAATCTCT

AAATACATTACTTCGTTCAAGACTGTATCTGCGAATCCAACGTTATCTTCAATCAGCATTGCTGACGGTG

CAGTTAACGTTGACCGTTCTAAAACAATTACAATTGAATTCAGCGATTCAGTTCCAAACCCAACAATCAC

TCTTAAGAAGGCTGACGGAACTTCATTTACTAATTACACTTTAGTAAATGTAAATAATGAAAATAAAACA

TACAAAATTGTATTCCACAAAGGTGTAACACTTGACGAGTTTACTCAATATGAGTTAGCAGTTTCAAAAG

ATTTTCAAACTGGTACTGATATTGATAGCAAAGTTACATTCATCACAGGTTCTGTTGCTACTGACGAAGT

AAAACCTGCTCTAGTAGGCGTTGGTTCATGGAATGGAACAAGCTATACTCAGGATGCTGCAGCAACACGA

CTTCGGTCTGTAGCTGACTTCGTTGCGGAGCCAGTTGCCCTTCAATTCTCAGAAGGTATCGATTTAACGA

ATGCAACTGTGACAGTAACAAATATTACTGATGATAAAACTGTTGAAGTTATTTCAAAAGAGAGTGTAGA

CGCAGACCATGATGCAGGTGCTACTAAGGAGACATTAGTAATTAACACAGTTACTCCTTTAGTACTTGAT

AACAGCAAGACTTATAAGATTGTTGTAAGTGGAGTTAAAGATGCAGCAGGTAATGTTGCAGATACTATTA

CATTCTATATTAAGTAATCTGGGCTAGGTGTTTGTCACCGCTCAAGGTTGTCAAAATATGTCGAAAAGCT

CTGCGGAGAGAAATCTCTGCGGGGCTTTTCTTTTTGCTCAAATCTGTATCGCGGCCGC

>gi|33767656|gb|AR360590.1| Sequence 13 from patent US 6596510

ATGAAAATAAAAACAGGTGCACGCATCCTCGCATTATCCGCATTAACGACGATGATGTTTTCCGCCTCGG

CTCTCGCCAAAATCGAAGAAGGTAAACTGGTAATCTGGATTAACGGCGATAAAGGCTATAACGGTCTCGC

TGAAGTCGGTAAGAAATTCGAGAAAGATACCGGAATTAAAGTCACCGTTGAGCATCCGGATAAACTGGAA

GAGAAATTCCCACAGGTTGCGGCAACTGGCGATGGCCCTGACATTATCTTCTGGGCACACGACCGCTTTG

GTGGCTACGCTCAATCTGGCCTGTTGGCTGAAATCACCCCGGACAAAGCGTTCCAGGACAAGCTGTATCC

GTTTACCTGGGATGCCGTACGTTACAACGGCAAGCTGATTGCTTACCCGATCGCTGTTGAAGCGTTATCG

CTGATTTATAACAAAGATCTGCTGCCGAACCCGCCAAAAACCTGGGAAGAGATCCCGGCGCTGGATAAAG

AACTGAAAGCGAAAGGTAAGAGCGCGCTGATGTTCAACCTGCAAGAACCGTACTTCACCTGGCCGCTGAT

TGCTGCTGACGGGGGTTATGCGTTCAAGTATGAAAACGGCAAGTACGACATTAAAGACGTGGGCGTGGAT

AACGCTGGCGCGAAAGCGGGTCTGACCTTCCTGGTTGACCTGATTAAAAACAAACACATGAATGCAGACA

CCGATTACTCCATCGCAGAAGCTGCCTTTAATAAAGGCGAAACAGCGATGACCATCAACGGCCCGTGGGC

ATGGTCCAACATCGACACCAGCAAATTGAATTATGGTGTAACGGTACTGCCGACCTTCAAGGGTCACCCA

TCCAAACCGTTCGTTGGCGTGCTGAGCGCAGGTATTAACGCCGCCAGTCCGAACAAAGAGTTGGCGAAAG

AGTTCCTCGAAAACTATCTGCTGACTGATGAAGGTCTGGAAGCGGTTAATAAAGACAAACCGCTGGGTGC

CGTAGCGCTGAAGTCTTACGAGGAAGAGTTGGCGAAAGATCCACGTATTGCCGCCACCATGGAAAACGCC

CAGAAAGGTGAAATCATGCCGAACATCCCGCAGATGTCCGCTTTCTGGTATGCCGTGCGTACTGCGGTGA

TCAACGCCGCCAGCGGTCGTCAGATCGTCGATGAAGCCCTGAAAGACGCGCAGACTAATTCGAGCTCGAA

CAACAACAACAATAACAATAACAACAACCTCGGGATCGAGGGAAGGATTTCAGAATTCGGATCCGCAAGC

TTCACAGATGTTGCGCCGCAATATAAAGATGCGATCGATTTCTTAGTATCAACTGGTGCAACAAAAGGTA

AAACAGAAACAAAATTCGGCGTTTACGATGAAATCACTCGTCTAGATGCGGCAGTTATTCTTGCAAGAGT

ATTAAAACTAGACGTTGACAACGCAAAAGACGCAGGCTTCACAGATGTGCCAAAAGACCGTGCAAAATAC

GTCAACGCGCTTGTAGAAGCTGGCGTATTAAACGGTAAAGCACCTGGCAAATTTGGTGCATACGACCCAT

TAACTCGCGTTGAAATGGCAAAAATCATCGCGAACCGTTACAAATTAAAAGCTGACGATGTAAAACTTCC

ATTCACTGATGTAAACGATACATGGGCACCATACGTAAAAGCGCTTTATAAATACGAAGTAACAAAAGGT

AAAACACCAACAAGCTTCGGTGCATACCAAAACATCACTCGCGGTGACTTTGCGCAATTTGTATATAGAG

CGGTGAATATTAATGCAGTGCCAGAAATAGTTGAAGTAACTGCGGTTAATTCGACTACAGTGAAAGTAAC

ATTCAATACGCAAATTGCTGATGTTGATTTCACAAATTTTGCTATCGATAACGGTTTAACTGTTACTAAA

GCAACTCTTTCTCGTGATAAAAAATCCGTAGAGGTTGTGGTAAATAAACCGTTTACTCGTAATCAGGAAT

ATACAATTACAGCGACAGGCATTAAAAATTTAAAAGGCGAGACCGCTAAGGAATTAACTGGTAAGTTTGT

TTGGTCTGTTCAAGATGCGGTAACTGTTGCACTAAATAATAGTTCGCTTAAAGTTGGAGAGGAATCTGGT

TTAACTGTAAAAGATCAGGATGGCAAAGATGTTGTAGGTGCTAAAGTAGAACTTACTTCTTCTAATACTA

ATATTGTTGTAGTTTCAAGTGGCGAAGTATCAGTATCTGCTGCTAAAGTTACAGCTGTAAAACCGGGAAC

AGCTGATGTTACTGCAAAAGTTACATTACCAGATGGTGTTGTACTAACAAATACATTTAAAGTGACAGTT

ACAGAAGTGCCTGTGCAAGTACAAAATCAAGGATTTACTTTAGTTGATAATCTTTCTAATGCTCCACAGA

ATACAGTTGCATTTAACAAAGCTGAGAAAGTAACTTCAATGTTTGCTGGAGAAACTAAAACAGTTGCAAT

GTATGATACTAAAAACGGTGATCCTGAAACTAAACCTGTTGATTTCAAAGATGCAACTGTACGTTCATTA

AATCCAATTATTGCAACAGCTGCTATTAATGGTAGTGAGCTCCTTGTCACAGCTAATGCTGGCCAATCTG

GAAAAGCTTCATTTGAAGTAACATTTAAAGATAATACAAAAAGAACATTTACAGTTGATGTGAAAAAAGA

CCCTGTATTACAAGATATTAAAGTAGATGCAACTTCTGTTAAACTTTCCGATGAAGCTGTTGGCGGCGGG

GAAGTTGAAGGAGTTAACCAAAAAACGATTAAAGTAAGTGCAGTTGACCAATACGGTAAAGAAATTAAAT

TTGGTACAAAAGGTAAAGTTACTGTTACAACTAATACAGAAGGACTAGTTATTAAAAATGTAAATAGCGA

TAATACAATTGACTTTGATAGCGGCAATAGTGCAACTGACCAATTTGTTGTCGTTGCAACAAAAGACAAA

ATTGTCAATGGTAAAGTAGAAGTTAAATATTTCAAAAATGCTAGTGACACAACACCAACTTCAACTAAAA

CAATTACTGTTAATGTAGTGAATGTAAAAGCTGACGCTACACCAGTAGGATTAGATATTGTAGCACCTTC

TGAAATTGATGTGAATGCTCCAAACACTGCTTCTACTGCAGATGTTGATTTTATTAATTTCGAAAGTGTT

GAGATTTATACACTCGATTCTAATGGTAACCGTCTTAAAAAAGTTACTCCAACTGCAACTACACTTGTAG

GTACTAATGATTATGTTGAAGTTAATGGGAATGTATTACAATTCAAGGGTAACGATGAATTAACGCTATT

AACTTCTTCTAGTACAGTAAACGTTGATGTAACAGCTGATGGAATTACAAAACGTATTCCAGTAAAATAT

ATCAACTCTGCAAGTGTACCTGCCAGTGCAACAGTAGCAACAAGTCCTGTTACTGTTAAGCTTAATTCAA

GTGATAATGATTTAACATTTGAAGAATTAATATTCGGTGTAATTGACCCTACACAATTAGTCAAAGATGA

AGACATCAACGAATTTATTGCAGTTTCAAAAGCGGCTAAAAATGATGGATATTTGTATAATAAACCGCTT

GTAACGGTTAAAGATGCATCAGGAAAAGTTATTCCAACAGGTGCAAATGTTTACGGTCTAAATCATGATG

CAACTAACGGAAACATTTGGTTTGATGAGGAACAAGCTGGCTTAGCTAAAAAATTTAGTGATGTACATTT

TGATGTTGATTTTTCATTAGCTAACGTTGTAAAAACTGGTAGCGGTACAGTTTCTTCATCGCCATCATTA

TCTGACGCAATTCAACTTACTAATTCAGGCGATGCAGTATCGTTTACATTAGTTATCAAATCAATTTATG

TTAAAGGCGCAGATAAAGATGATAATAACTTACTTGCAGCCCCTGTTTCTGTCAATGTGACTGTGACAAA

ATAATTTTGAGGTTCGGTCTCTGTTACCATTTGAAAAATGCCGAAAAGCTCTGCGGAGAGAAATCTCTGC

GGGGCTTTTCTTTTTGGTTCTATGTCAATTGTTGAGGTGCATGGATCCTCTAGAGTCGACCTGCAGGCAA

GCTTG
